# Natural carrier-free self-assembled binary polyphenol nanoparticles remodel the gut microenvironment for inflammatory bowel disease prevention

**DOI:** 10.1016/j.mtbio.2026.103063

**Published:** 2026-03-28

**Authors:** Qiwen Xie, Huan Xu, Xiaoming Yang, Ying Chen, Zhenjiang Zech Xu

**Affiliations:** a State Key Laboratory of Food Science and Resources, Nanchang University, Nanchang, 330047, China; b Shenzhen Hospital, Southern Medical University, Shenzhen, 518000, China

**Keywords:** Carrier-free nanoparticles, Self-assembly, Polyphenols, Inflammatory bowel disease, Gut microbiota, Immune modulation

## Abstract

Developing biocompatible, multi-target therapeutics remains a critical challenge in the management of inflammatory bowel disease (IBD). Herein, we engineered a carrier-free nanoplatform (Cur-Ant NPs) via the facile self-assembly of two natural polyphenols: curcumin (Cur) and anthocyanin (Ant). Spectroscopic analysis and molecular dynamics simulations confirmed that the assembly is stabilized by robust π-π stacking and hydrogen bonding networks, yielding uniform, spherical nanostructures with integrated functionality. In a dextran sulfate sodium (DSS)-induced colitis model, orally administered Cur-Ant NPs demonstrated superior therapeutic efficacy compared to both free polyphenols and the clinical standard, sulfasalazine (SASP). The nanoparticles' potent anti-inflammatory activity was initially validated in a zebrafish model, where they effectively inhibited neutrophil infiltration and scavenged reactive oxygen species (ROS). These protective effects were further substantiated in a murine model, where multi-omics analysis revealed a tripartite mechanism of action: reinforcing the intestinal epithelial barrier, mitigating pro-inflammatory cytokine responses, and remodeling the dysbiotic gut microbiome. Our findings establish Cur-Ant NPs as a potent, safe candidate for IBD prevention and highlight a scalable, green engineering strategy for designing next-generation nanomedicines based on the supramolecular co-assembly of natural bioactive agents.

## Highlights

• **Carrier-free nanomedicine** engineered via **green supramolecular self-assembly** of binary natural polyphenols.
• **Molecular dynamics simulations** and spectral analysis elucidate the assembly mechanism driven by π-π stacking and H-bonding.
• Cur-Ant NPs demonstrate superior **colonic accumulation** and therapeutic efficacy over **sulfasalazine** in colitis models.
• The nanoplatform synergistically remodels the **gut microenvironment** by scavenging ROS, restoring barrier integrity, and reversing dysbiosis.

## Graphical abstract

**Figure:**
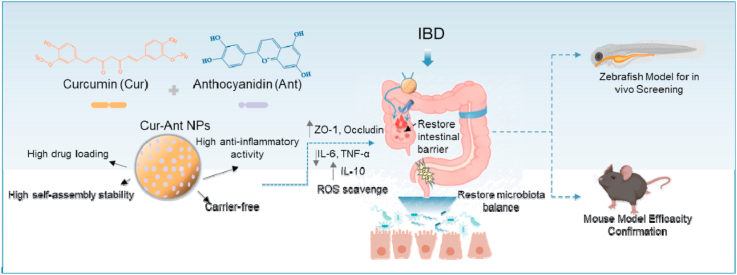

## 1 Introduction

Inflammatory bowel disease (IBD), a relentless inflammatory condition of the gut, now affects millions worldwide [[1], [2], [3]]. Its rapidly rising incidence in newly industrialized nations makes it an increasingly common and formidable global health challenge [1,[4], [5], [6]]. Despite extensive research, the precise cause of IBD remains unclear [[7], [8], [9]], and its onset is widely attributed to a complex interplay among genetic susceptibility, environmental factors, gut microbiota disturbances, and host immune dysregulation [[10], [11], [12], [13]]. Ultimately, this multifactorial pathogenesis disrupts colonic homeostasis [9], leading to severe intestinal barrier dysfunction [7,13]. This results in a cascade of debilitating symptoms including weight loss, abdominal pain, and hematochezia, which severely impair quality of life [5,12,14]. The complex etiology and clinically refractory nature of IBD present a formidable challenge in modern medicine.

Within the complex pathogenic network of IBD, the massive accumulation of reactive oxygen species (ROS) serves as a critical node that amplifies a vicious cycle of oxidative stress and inflammation [15,16]. This process begins as ROS drive macrophage polarization toward a pro-inflammatory M1 phenotype, triggering a wave of cytokines (e.g., TNF-α, IL-6) and the massive infiltration of neutrophils, which further release cytotoxic proteases and ROS to exacerbate mucosal damage [[17], [18], [19], [20], [21], [22]]. This persistent oxidative-inflammatory milieu inflicts devastating damage upon the intestinal epithelial barrier and gut microbiota—the two cornerstones of intestinal health [14]. Specifically, inflammatory mediators suppress tight junction proteins, increasing permeability and facilitating the translocation of luminal pathobionts, which subsequently induces profound gut dysbiosis [13,14,[23], [24], [25], [26], [27]]. Consequently, neutralizing excess ROS to disrupt this self-amplifying cycle and targeting the pathological “barrier-microbiota” axis have emerged as paramount therapeutic strategies for mitigating IBD progression [12,28].

The current clinical armamentarium for IBD relies on an expanding repertoire of drugs, from conventional 5-aminosalicylic acid and corticosteroids to modern immunosuppressants and biologics, notably *anti*-TNF-α agents [7,9,12,29,30]. Although these therapies demonstrate considerable efficacy in inducing remission, promoting mucosal healing, and reducing the risk of colorectal neoplasia, their benefits are offset by substantial associated costs [10]. Primarily acting as broad anti-inflammatory or immunosuppressive agents, their efficacy is often shadowed by a spectrum of serious side effects, including gastrointestinal distress and immune dysfunction [13]. Critically, this therapeutic paradigm largely targets the downstream inflammatory cascade, often failing to comprehensively address the core pathological pillars of barrier dysfunction and microbial dysbiosis described earlier [27,31,32]. Consequently, a substantial portion of patients either lose response over time or are refractory to treatment from the outset, ultimately requiring surgical intervention [33]. This pervasive therapeutic ceiling emphasizes a critical unmet clinical need in IBD management. There is, therefore, an urgent imperative to pioneer a new therapeutic paradigm: one that leverages safe, food-grade natural ingredients to develop a desirable, sustainable, and cost-effective strategy for treating IBD through multi-target action.

In the burgeoning field of natural product therapeutics, food-derived polyphenols have emerged as a premier strategy for the holistic management of IBD. This prominence is attributed to their exceptional safety profiles, cost-effectiveness, and multifaceted regulatory capacity across the intestinal mucosal barrier, immune homeostasis, and microbial ecology [34]. Within this pharmacological landscape, Cur and Ant represent a compelling therapeutic duo with highly complementary bioactivities [35]. Cur is a preeminent anti-inflammatory phytochemical, renowned for its ability to modulate the core transcriptional machinery of inflammation, specifically by suppressing the NF-κB and MAPK signaling cascades [17,36]. In contrast, Ant offers a superior antioxidant defense; its unique catechol and pyrogallol moieties enable the direct and potent scavenging of ROS [37,38]. We hypothesized that their co-administration would yield a potent synergistic effect, establishing a dual-layered defense that simultaneously neutralizes upstream oxidative stress and dampens downstream pro-inflammatory signaling.

Despite this promise, the clinical translation of such polyphenolic combinations remains hindered by a “bioavailability bottleneck.” Both Cur and Ant exhibit extreme susceptibility to environmental stressors—including light, thermal fluctuations, and gastrointestinal pH shifts—leading to rapid degradation and poor systemic absorption [39,40]. This fundamental limitation severely constrain their therapeutic applicability and underscores the critical need for innovative delivery strategies. To transcend these biopharmaceutical constraints, nanotechnology has emerged as a transformative frontier [36,41]. However, traditional carrier-based delivery systems frequently encounter “translational hurdles,” including low drug-loading efficiency and potential long-term systemic toxicity stemming from synthetic excipients [9]. In this context, the carrier-free self-assembly paradigm represents a sophisticated “minimalist” alternative. This bottom-up approach leverages the intrinsic molecular recognition of therapeutic agents to architect stable nanostructures via a delicate interplay of non-covalent forces, such as π-π stacking and hydrogen bonding [[42], [43], [44]]. Recent breakthroughs in “Supramolecular Chinese Medicine” have demonstrated the viability of this strategy [45]; for instance, natural alkaloids like berberine have been successfully co-assembled with phenolic acids or lignans to form nanomedicines with enhanced colonic biodistribution and synergistic efficacy [43,46].

**Scheme 1:**
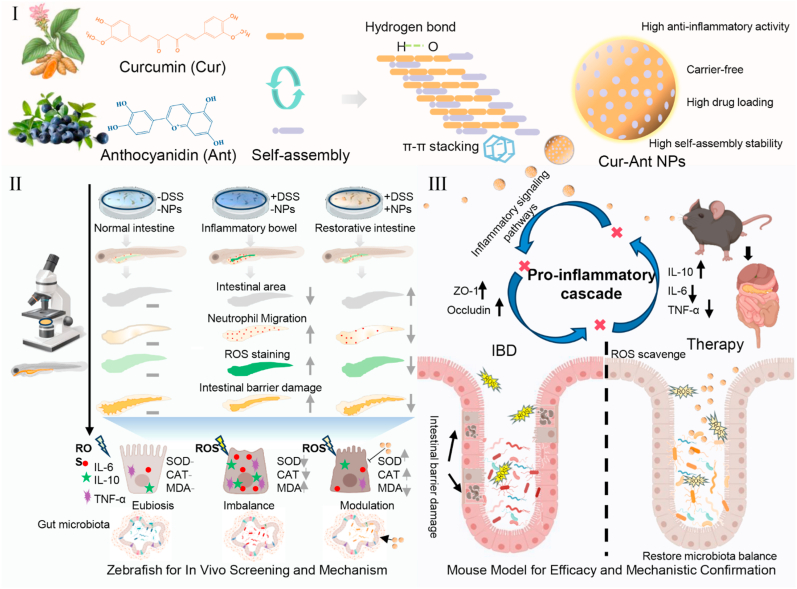
Carrier-Free Cur-Ant Co-Assembled Nanoparticles via Oral Administration Alleviate Inflammatory Bowel Disease in Zebrafish and Mice through Antioxidant, Immunomodulatory, and Microbiota-Modulating Effects.

Inspired by these principles of natural supramolecular chemistry, herein, we report the design and synthesis of novel, carrier-free Cur-Ant NPs. We hypothesized that the complementary aromatic profiles of Cur and Ant could drive their spontaneous co-assembly into highly stable nanoparticles capable of surviving the hostile gastrointestinal environment. Our results demonstrate that these NPs exhibit superior stability and targeted colonic accumulation compared to their free forms. Mechanistically, we reveal that Cur-Ant NPs restore intestinal homeostasis by executing a multi-pronged therapeutic program: concurrently scavenging mucosal ROS, suppressing macrophage-driven inflammatory storms, reinforcing the epithelial barrier, and favorably remodeling the gut microbiota (Scheme 1). This study not only offers a potent, biosafe intervention for IBD but also validates the potential of polyphenolic self-assembly as a versatile platform for synergistic multi-target management.

## 2 Experimental section

### 2.1 Materials and reagents

Cur (458-37-7), Ant (13,306-05-3), 1,1-diphenyl-2-picrylhydrazyl (DPPH), 2,2′-azino-bis (3-ethylbenzothiazoline-6-sulfonic acid (ABTS) and 3,3′,5,5′-tetramethylbenzidine were supplied by Shanghai Macklin Biochemical Technology Co., Ltd. Sulfasalazine (SASP) was purchased from Puxitang Biotechnology Co., Ltd. (Beijing, China). Dextran Sulfate Sodium (DSS, 40 kDa) was obtained from MP Biomedicals LLC. H_2_O_2_ (30%) was acquired from Chengdu Jinshan Chemical Reagent Co. Ltd. For more information on materials and reagents, please refer to the Supporting Information. Absolute ethyl alcohol, dimethyl sulfoxide and NaOH (99%) was acquired from Kelong Chemical Reagent Co., Ltd. (Chengdu, China). All chemical reagents were used without further purification. The following test kits were purchased from Nanjing Jiancheng Bioengineering Institute Co., Ltd.: Total Superoxide Dismutase (T-SOD) Assay Kit (Hydroxylamine method), Catalase (CAT) Assay Kit (Visible light method), Malondialdehyde (MDA) Assay Kit (TBA method), ROS Assay Kit, and Total Protein (TP) Assay Kit.

### 2.2 Preparation of Cur-Ant NPs

Cur-Ant NPs were fabricated via self-assembly, a process predominantly driven by hydrophobic interactions, π−π stacking, and hydrogen bonding. Briefly, curcumin and anthocyanin were dissolved in a mixture of 6 mL dimethyl sulfoxide (DMSO) and 4 mL ultrapure water at varying molar ratios (Cur:Ant = 2:1, 1:1, 1:2, 1:3, and 1:4). The resultant solution was subsequently heated to 70 °C and subjected to continuous ultrasonication for 5 min. Finally, the aforementioned products were purified by dialysis against water (MWCO 3500 Da) for 48 h, followed by lyophilization to yield Cur-Ant NPs with distinct curcumin-to-anthocyanin proportions.

### 2.3 Characterization of NPs

Scanning electron microscopy (SEM) imaging was performed using a Hitachi S-4800 field-emission scanning electron microscope. Dynamic light scattering (DLS) and zeta potential measurements were conducted on a Malvern Panalytical Nano ZS90 instrument (Malvern, UK). Fluorescence spectra were acquired with an FLS1000 spectrofluorometer (Edinburgh Instruments, UK). UV-vis absorption spectra were recorded on a Hitachi UH4150 UV-vis-NIR spectrophotometer (Japan). X-ray diffraction (XRD) patterns were collected on a Thermo Scientific ESCALAB 250Xi + X-ray diffractometer. Fourier transform infrared (FTIR) spectroscopy was carried out using a Nicolet iS50 FTIR spectrometer (Thermo Fisher Scientific). ^1^H nuclear magnetic resonance (NMR) spectra were obtained on a JEOL JNM-ECZ600R NMR spectrometer (600 MHz). UHPLC Q-Exactive Orbitrap HRMS: UHPLC Q-Exactive Orbitrap HRMS was performed on a QExactivePlus (Thermo Fisher Scientific, US), and the results were obtained in the positive ion mode.

### 2.4 Molecular dynamics simulation

Molecular dynamics (MD) simulations were performed to investigate the interactions between Cur and Ant. Two systems with different molecular ratios were constructed in a 5 × 5 × 5 nm cubic box: one with 10 Cur and 10 Ant molecules, and the other with 5 Cur and 15 Ant molecules. The detailed methodology and parameters for the simulations are described in the Supporting Information.

### 2.5 ABTS radical scavenging assay

The total antioxidant capacity of Cur-Ant NPs was evaluated using the ABTS assay. Specifically, a mixture of 7.4 mM ABTS and 2.6 mM ammonium persulfate (1:1, v/v) was stored in the dark at 4 °C overnight to generate ABTS+• radicals. The stock solution was then diluted with PBS until the absorbance at 734 nm reached 0.78 ± 0.05 to obtain the ABTS working solution. After that, 100 μL of the ABTS working solution was added to 200 μL of various concentrations (5, 10, 15, 20, and 30 μg/mL) of Cur-Ant NPs, and the UV absorbance at 734 nm was monitored every 5 min for 30 min. Furthermore, in order to compare the differences in ABTS free radical scavenging among free Cur, Ant and different nanoparticles. 100 μL 20 μg/mL samples (including free Cur and Cur-Ant (1:1), Cur-Ant (1:2), Cur-Ant (1:3) and Cur-Ant (1:4) NPs were added. Finally, the absorbance value of the above mixed solution at 734 nm was measured at 20 min.

### 2.6 DPPH free radical scavenging activity

For DPPH assay testing, a series of aqueous solutions of Cur-Ant NPs with concentrations ranging from 0 to 30 μg/mL were prepared. Subsequently, 200 μL of these solutions were added to an ethanol solution of DPPH (200 μg/mL, 100 μL). The UV−vis spectrophotometer (LAMBDA 650, PerkinElmer) was used to test the absorbance value of the above mixed solution at 517 nm at different time (up to 30 min). Moreover, in order to compare the differences in DPPH free radical scavenging among free Cur, Ant and different NPs. 100 μL 20 μg/mL samples (including free Cur, Ant, Cur-Ant (1:1), Cur-Ant (1:2), Cur-Ant (1:3) and Cur-Ant (1:4) NPs were added. Finally, the absorbance value of the above mixed solution at 734 nm was measured at 20 min.

### 2.7 ·OH scavenging assay

1 mM TMB was prepared with DMSO, 5 mM FeSO_4_-7H_2_O was prepared with HAc-NaAc buffer, and 50 mM H_2_O_2_ aqueous solution was prepared. Then, a series of aqueous of Cur-Ant at concentrations of 0, 10, 20, 30, 40 and 50 μg/mL were prepared. The hydroxyl radical (•OH) scavenging capacity was assessed as follows: A 1.5 mL microcentrifuge tube was sequentially loaded with 500 μL of the Fenton reaction mixture, 100 μL of TMB solution, and 200 μL of sample solutions at gradient concentrations. After incubation at room temperature for 10 min, the absorbance at 652 nm was quantified using a microplate reader to determine the extent of •OH elimination.

### 2.8 Superoxide anion radical scavenging activity

The superoxide anion radical scavenging capacity of the samples was evaluated using the photochemical riboflavin-methionine-nitroblue tetrazolium (NBT) system. The assay is based on the principle that superoxide radicals (O_2_^•–^), generated by the reaction of riboflavin and methionine under light illumination, reduce NBT to form a blue formazan pigment, the absorbance of which can be suppressed by the presence of scavenging agents. All reagents were prepared in 0.05 M phosphate buffer (pH 7.8). To establish a concentration-dependent profile, the test samples were incorporated into the reaction mixture to achieve final concentrations of 5, 10, 15, 20, and 30 μg/mL. The reaction was conducted in a total volume of 3 mL, maintaining final concentrations of 1.33 × 10^−5^ M riboflavin, 4.46 × 10^−5^ M methionine, and 8.15 × 10^−8^ M NBT. The mixtures were placed under 20 W fluorescent lamps and illuminated at 25 °C for 40 min to induce the photochemical reaction. After incubation, the absorbance of the solution was measured at 560 nm using a spectrophotometer, where a decrease in absorbance relative to the control indicated enhanced scavenging activity. The percentage of superoxide anion scavenged was calculated using the equation:

$$O_{2}^{\cdot –}\;\text{scavenging}\;\left(\%\right)=\left[1-\left(A_{S}\;/\;A_{C}\right)\right]\times 100$$

where A_C_ is the absorbance of the control and A_S_ is the absorbance in the presence of curcumin or standards.

### 2.9 In vivo efficacy against intestinal inflammation in a zebrafish model

At 3 days post-fertilization (dpf), AB strain zebrafish or transgenic Tg (*lyz:DsRed*2) larvae were randomly divided into six groups (n = 30 per group): a control group, a 0.4% DSS model group, and four treatment groups receiving 0.4% DSS co-administered with either SASP, free Ant, free Cur, or Cur-Ant NPs. The concentration for all drug treatments was maintained at 500 μg/l. After the treatment period, larvae were collected for a comprehensive evaluation of colitis mitigation. The analyses performed included histopathological examination of the intestine via Hematoxylin and Eosin (H&E) staining, quantification of intestinal immune cell (neutrophil) infiltration, and in vivo imaging of ROS. Furthermore, the expression levels of inflammation-related genes were assessed by quantitative real-time PCR (qPCR; primer sequences are provided in Supplementary Table S1), and key oxidative stress biomarkers (SOD, CAT, and MDA) were measured. Finally, the composition of the intestinal microbiota was analyzed using 16 S rRNA gene sequencing. Detailed methodologies for zebrafish husbandry, preliminary nanoparticle toxicity assays, and all specific analytical procedures are described in the Supporting Information.

### 2.10 In vivo therapeutic efficacy in a murine colitis model

All animal experiments were approved by the Animal Ethics Committee of Nanchang University [SYXK (Gan) 2021–0004]. To evaluate the biological activity of Cur-Ant NPs in vivo, mice received dextran sodium sulfate (DSS, 40 kDa, MP Biomedical) in their drinking water to induce IBD during 7 consecutive days. A total of 48 male C57BL/6 mice were randomly allocated into six groups (n = 8 per group): (1) Control, (2) DSS, (3) DSS + SASP, (4) DSS + Free Ant, (5) DSS + Free Cur, and (6) DSS + Cur - Ant NPs groups. The experimental protocol comprised a 3-day prophylactic pre-treatment phase followed by a 7-day concomitant DSS-induction/treatment phase. From Day 1 to Day 3, mice in all treatment groups (Groups 3–6) were administered their respective formulations daily via oral gavage at a dosage of 10 mg/kg (resuspended in 200 μL PBS per mouse). Simultaneously, the Control and DSS groups received equal volumes of PBS (0.01 M, pH 7.4). Starting from Day 4, acute colitis was induced in all mice except the Control group by replacing their drinking water with 2.5% (w/v) dextran sulfate sodium (DSS, 40 kDa) for 7 consecutive days. Throughout this 7-day period, the daily oral gavage of the designated drugs was continued to assess their therapeutic efficacy [12,42]. Disease progression was monitored daily by DAI according to the criteria provided in Supplementary Table S2. At the end of the experiment, colon tissues and fecal samples were collected for a comprehensive evaluation of therapeutic efficacy. The analyses included histological and histochemical staining (H&E and AB-PAS) (scoring criteria in Supplementary Table S3), quantification of inflammatory cytokines (TNF-α, IL-6, IL-10) by ELISA, and assessment of intestinal barrier integrity through immunofluorescence and Western blot analysis of tight junction proteins (ZO-1 and OCCLUDIN). Furthermore, the accumulation of ROS and the expression of myeloperoxidase (MPO) in colon tissues were evaluated via immunofluorescence and immunohistochemical (IHC) staining, respectively, to assess the antioxidant and anti-inflammatory capacity of the treatments. Detailed procedures for animal care, specific experimental protocols, and all bioinformatic analysis pipelines are provided in the Supporting Information.

### 2.11 RNA extraction, library construction, and sequencing

Total RNA was extracted from murine colon tissues using TRIzol® Reagent (Invitrogen, Carlsbad, CA, USA). The integrity of the RNA was verified, and only high-quality samples (RNA Integrity Number, RIN ≥8.0) were used for library preparation. Library construction and sequencing were performed by Novogene Co., Ltd. (Beijing, China). Briefly, mRNA was enriched and prepared into sequencing libraries using the TruSeq Stranded mRNA Library Prep Kit (Illumina, San Diego, CA, USA). The libraries were then sequenced on an Illumina NovaSeq 6000 platform to generate 150 bp paired-end reads. Detailed procedures for RNA quality control and library construction are provided in the Supplementary Information.

### 2.12 16 S rRNA gene sequencing and analysis

Fecal microbial genomic DNA was extracted using the E. Z.N.A.® Soil DNA Kit (Omega Bio-tek, Norcross, GA, USA). The V3-V4 hypervariable regions of the 16 S rRNA gene were amplified using the primer pair 338 F/806 R. Paired-end (2 × 250 bp) sequencing was performed on an Illumina NovaSeq 6000 platform by Novogene Co., Ltd. (Beijing, China). Bioinformatic analysis was conducted using QIIME 2. Raw sequences were processed with the DADA2 plugin to generate an amplicon sequence variant (ASV) feature table. Taxonomy was assigned against the SILVA database (v138). Alpha and beta diversity analyses were performed, and statistical significance between groups was assessed by Permutational Multivariate Analysis of Variance (PERMANOVA). Differentially abundant taxa were identified using Linear discriminant analysis effect size (LEfSe), and their correlations with host inflammatory markers were evaluated using Spearman's rank correlation. Detailed protocols are available in the Supplementary Information.

### 2.13 In Vivo Biodistribution and colonic retention study

To investigate the gastrointestinal transit behavior and colonic targeting capability of the nanoplatform, the near-infrared lipophilic fluorescent dye 1,1-dioctadecyl-3,3,3,3-tetramethylindotricarbocyanine iodide (DiR) was encapsulated within the Cur-Ant NPs (DiR@Cur-Ant NPs) to serve as a fluorescent tracer.

**Preparation of DiR-loaded Nanoparticles:** DiR@Cur-Ant NPs were fabricated using a co-assembly method adapted from the preparation of blank nanoparticles. Briefly, DiR was co-dissolved with Cur and Ant in the mixed solvent of dimethyl sulfoxide (DMSO) and ultrapure water (6:4, v/v) to obtain a theoretical DiR concentration of 500 μg/mL. The mixture was heated to 70 °C and subjected to continuous ultrasonication for 5 min to facilitate self-assembly. To remove the organic solvent and unloaded free DiR, the resulting solution was dialyzed against distilled water (MWCO 3500 Da) for 48 h in the dark, followed by lyophilization to obtain DiR@Cur-Ant NPs.

**In Vivo Imaging:** Male C57BL/6 mice were randomly divided into two groups: the Free DiR group and the DiR@Cur-Ant NPs group. The mice were fasted overnight with free access to water prior to the experiment. The DiR@Cur-Ant NPs and Free DiR (dissolved in 1% DMSO/water) were orally administered to the mice via gavage at an equivalent DiR dose. At predetermined time points (3, 6, 12, and 24 h) post-administration, the mice were anesthetized, and the fluorescence distribution was visualized using an IVIS Spectrum imaging system (PerkinElmer). The imaging parameters were set with an excitation wavelength of 760 nm and an emission wavelength of 790 nm.

**Ex Vivo Imaging:** To precisely evaluate the retention of nanoparticles in the gastrointestinal tract, the mice were sacrificed immediately after the 24 h imaging point. The entire gastrointestinal tracts (including the stomach, small intestine, and colon) were harvested. The ex vivo fluorescence images were captured under the same parameters as the in vivo imaging. The fluorescence intensity in the region of interest (ROI) of the colon was quantified to assess the colonic targeting efficiency.

### 2.14 Biosafety assessment

To evaluate the hemocompatibility of the nanoparticles, a hemolysis assay was performed using fresh rat whole blood. Erythrocytes (Red blood cells, RBCs) were isolated by centrifugation and washed three times with 0.9% NaCl solution (saline). A stock suspension of RBCs was then prepared by diluting the packed cells with saline. For the assay, 0.5 mL of the RBC suspension was mixed with 0.5 mL of saline containing different concentrations of Cur-Ant NPs. Samples of RBCs mixed with 0.5 mL of saline and 0.5 mL of deionized water served as the negative and positive controls, respectively. All mixtures were incubated in a 37 °C water bath for 2 h. After incubation, the samples were centrifuged to pellet the intact RBCs. The absorbance of the resulting supernatant, corresponding to the amount of released hemoglobin, was measured at 541 nm using a UV-visible spectrophotometer. The hemolysis percentage was calculated using the following formula [47]:

$$\text{Hemolysis}\;\left(\%\right)=\left({\text{OD}}_{\text{sample}}-{\text{OD}}_{\text{negative}}\right)\;/\;\left({\text{OD}}_{\text{positive}}-{\text{OD}}_{\text{negative}}\right)\times 100\%$$

The in vivo biocompatibility of Cur-Ant NPs was further evaluated in C57BL/6 mice. The animals were administered with an oral gavage of Cur-Ant NPs daily for a period of 10 days. At the end of the treatment, blood serum was analyzed for key liver (ALT, AST, TP/ALB, TBIL) and kidney (CREA, Urea) function markers [43]. Major organs (heart, liver, spleen, lungs, kidneys) were harvested for histopathological examination via H&E staining to assess potential organ damage [48].

### 2.15 Statistical analysis

Data are presented as the mean ± standard deviation (SD). All statistical analyses were conducted using IBM SPSS Statistics (Version 27.0, Armonk, NY, USA). Prior to analysis, data were assessed for normal distribution and homogeneity of variances. For comparisons between two independent groups, an unpaired Student's t-test was used. For comparisons among three or more groups, a one-way analysis of variance (ANOVA) was performed. Post-hoc comparisons were made using Fisher's least significant difference (LSD) test. A p-value less than 0.05 was considered statistically significant. Significance levels in figures are denoted as *p* < 0.05 (∗), *p* < 0.01 (∗∗), and *p* < 0.001 (∗∗∗) relative to the control group, unless otherwise stated. Specific details on the statistical tests and the number of replicates (n) are provided in the figure legends.

## 3 Results and discussion

### 3.1 Carrier-free Co-assembled Cur-Ant NPs

Polyphenolic natural products play a pivotal role in the advancement of intelligent drug delivery systems, serving both as primary building blocks for structural frameworks and as therapeutic agents in pharmaceutical formulations [[48], [49], [50]]. The development of innovative drug delivery platforms has been fundamentally driven by the necessity to preserve the inherent physicochemical stability of natural bioactive compounds. Here, it was demonstrated that Cur and Ant can be successfully co-assembled through a classical nanoprecipitation approach, generating nanostructures (nanoparticles or nanofibers) with tunable dimensions and morphologies by modulating their molar ratios. These structural variations reveal a remarkable correlation between compositional parameters and supramolecular organization patterns.

**Fig. 1:**
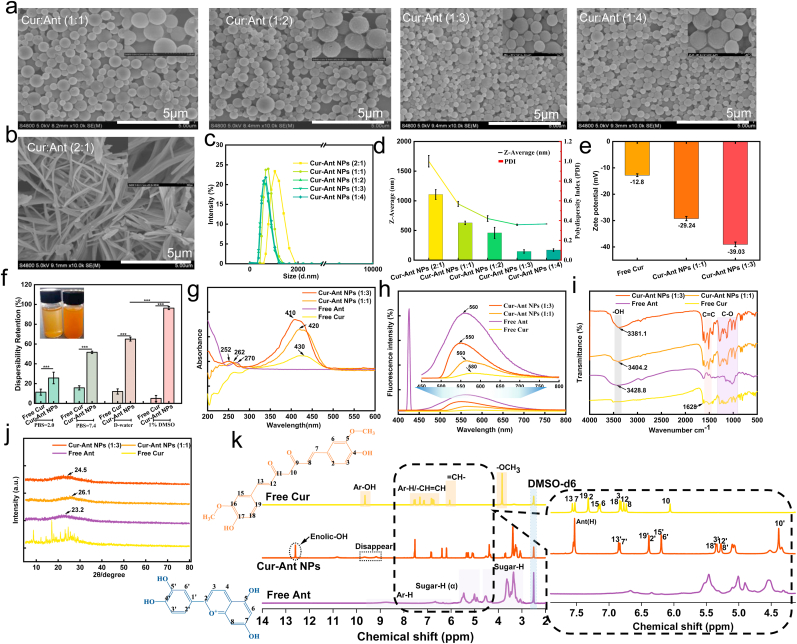
**Physicochemical Characterization and Elucidation of the Self-Assembly Mechanism of Cur-Ant Nanoparticles.** (a, b) Scanning electron microscopy (SEM) images of Cur-Ant nanostructures formed at different molar ratios: (a) spherical nanoparticles (Cur:Ant = 1:1, 1:2, 1:3, 1:4) and (b) nanofibers (Cur:Ant = 2:1). (c) Dynamic light scattering (DLS) analysis of the particle size distribution of Cur-Ant NPs. (e) Zeta potential of free Cur, Cur-Ant (1:1), and Cur-Ant (1:3) NPs. (f) Dispersibility of Cur and Cur-Ant NPs in different relevant media: PBS (pH 2.0), PBS (pH 7.4), deionized water (D-water), and 1% DMSO. (g) UV-vis absorption spectra of free Cur, free Ant, Cur-Ant (1:1) and Cur-Ant (1:3) NPs. (h) Fluorescence emission spectra of free Cur, free Ant, Cur-Ant (1:1) and Cur-Ant (1:3). (i) Fourier-transform infrared (FT-IR) spectra of the samples, indicating intermolecular interactions. (j) X-ray diffraction (XRD) patterns revealing the physical state of the samples. (k) ^1^H nuclear magnetic resonance (NMR) spectra of free Cur, free Ant, and Cur-Ant NPs in DMSO-*d*_6_, elucidating the molecular interaction sites during self-assembly.

Specifically, the morphology of the self-assembled Cur-Ant nanostructures was investigated using scanning electron microscopy (SEM), as shown in Fig. 1a–b. The molar ratio of Cur to Ant significantly dictated the resulting architecture, highlighting the critical influence of stoichiometric balance on the self-assembly pathway. The components self-organize into spherical nanostructures with a smooth and integrated surface when Ant exhibits equivalent or predominant molar composition in Cur-Ant NPs (Fig. 1a), whereas preferential molar dominance of Cur leads to an interconnected network of nanofibers (Fig. 1b). Notably, these nanospheres exhibited a high electron density and a distinctively solid, non-collapsing morphology under vacuum during SEM preparation, which is characteristic of dense solid-core matrices rather than hollow vesicular structures. This morphological transition is likely governed by the interplay of π-π stacking and hydrophobic interactions [46,51]; Cur's high hydrophobicity drives elongated growth, while the more hydrophilic Ant molecules act as supramolecular stabilizers that modulate interfacial curvature to favor spherical packing. Unlike amphiphilic molecules that form bilayers or vesicles, the planar aromatic structures of Cur and Ant facilitate continuous molecular stacking, leading to the formation of kinetically stable, solid supramolecular aggregates. Notably, the micromorphology of the free monomer molecules Ant and Cur was substantially different from that of the Cur-Ant NPs (Fig. S1a), confirming the successful supramolecular co-assembly of the two components. Moreover, a clear trend of decreasing particle size and improved monodispersity was observed with increasing Ant proportion, culminating in the Cur-Ant (1:3) formulation which presented as uniform, discrete nanospheres (Fig. 1a). As the Ant proportion increases, the enhanced surface charge density provides greater electrostatic repulsion, which effectively limits crystal growth and stabilizes the nanoparticles at a sub-micron scale.

Dynamic light scattering (DLS) analysis further quantified the size characteristics of the spherical nanoparticles (Fig. 1c–d). Consistent with SEM, the Z-average diameter decreased with increasing Ant content for the nanoparticle formulations, with Cur-Ant (1:3) exhibiting the smallest average diameter of approximately 596.1 nm and a low polydispersity index (PDI) of circa 0.09 (Fig. 1d). The combination of a high refractive index in DLS and the absence of indentation in SEM further corroborates the formation of rigid, dense nanoparticles. While conventional nanomedicines often prioritize particles <200 nm for systemic uptake, the sub-micron scale (∼600 nm) of Cur-Ant NPs is optimized for localized IBD treatment. This size range facilitates enhanced accumulation and sustained retention at the inflamed intestinal mucosa by exploiting the ‘EPR-like’ effect and mucoadhesive interactions, ensuring high local drug concentration at the lesion site while reducing systemic exposure [[52], [53], [54], [55]]. The zeta potential measurements (Fig. 1e) indicated that all nanoparticles possessed a negative surface charge, which became increasingly negative with higher Ant ratios (e.g., Cur: 12.8 mV; Cur-Ant (1:1): 20.24 mV; Cur-Ant (1:3): approx. −39 mV). The increase in anthocyanin ratio leads to a shift in zeta -potential towards more negative values, a phenomenon driven by the high density of phenolic hydroxyl groups. At near-neutral pH, the deprotonation of these groups at the nanoparticle interface significantly enhances the surface negative charge density, as widely validated in polyphenol-based self-assemblies [56]. Such high absolute zeta potential values suggest excellent colloidal stability due to strong inter-particle electrostatic repulsion [[57], [58], [59]]. To empirically verify this stability in physiological environments, the aqueous dispersibility of the optimal Cur-Ant (1:3) NPs was compared with free Cur (Fig. 1f). While free Cur rapidly sedimented due to its intrinsic hydrophobicity and tendency for π-π driven self-aggregation, the Cur-Ant NPs exhibited excellent suspension stability across various media, including PBS (pH = 2.0 and 7.4), deionized water, and 1% (v/v) DMSO. Notably, the dispersibility retention exhibited a distinct solvent-dependent characteristic, with the highest stability observed in the 1% (v/v) DMSO group (Fig. 1f). This suggests that the presence of a trace amount of co-solvent may further optimize the interfacial wetting of the nanoparticles and enhance the solvation of the Ant-based hydrophilic shell. Such an enhanced solvation layer effectively reinforces the steric hindrance and electrostatic repulsion, overriding the hydrophobic attraction of the Cur core and ensuring the robust colloidal integrity necessary for efficient gastrointestinal transit [60,61].

The structural integrity and gastrointestinal stability of Cur-Ant NPs were further validated by their distinct pH-responsive release profile (Fig. S1b), characterized by a highly suppressed release under acidic conditions (pH 2.0, simulating the gastric environment) and a significantly enhanced release at neutral pH (7.4, simulating the intestinal environment). This behavior demonstrates the robust stability of the nanoparticles against premature dissociation in Simulated Gastric Fluid, likely governed by the protonation state of the polyphenol network. In acidic environments, the phenolic hydroxyl groups remain protonated, promoting a tightly packed supramolecular structure via dense hydrogen bonding that effectively locks the cargo within the nanomatrix. Conversely, the transition to a neutral or slightly alkaline environment (representative of Simulated Intestinal Fluid, SIF) triggers deprotonation, increasing the surface negative charge density and subsequent inter-chain electrostatic repulsion, which facilitates structural swelling and site-specific colonic release [59,60]. These results collectively suggest that Cur-Ant NPs can successfully withstand gastric transit and selectively release their therapeutic payload upon reaching the colonic site [62,63].

In addition, storage stability studies demonstrated Cur-Ant NPs exhibited remarkable colloidal stability as demonstrated by the particle size and PDI of Cur-Ant NPs over 12 days, suggesting their excellent monodispersity and robust resistance against aggregation (Fig. S1c). Cur has low stability, which is easy to reduce or even lose biological activity by different external factors, such as high temperatures, UV radiation, and the nonacid environment [48]. Therefore, the effects of the temperature, UV exposure time, and pH on the stability of Cur-Ant NPs were evaluated. The retention rates of Cur-Ant NPs and free Cur were compared at different temperatures and with improved time under UV radiation and in buffer solution of pH = 9. It was found that free Cur had a faster decrease in retention rates compared to the Cur in Cur-Ant NPs at different temperatures (Fig. S1d), similar to UV exposure time (Fig. S1e) and pH = 9 environment (Fig. S1f). This enhanced stability is attributed to the protective ‘molecular cage’ formed by Ant through multi-valent hydrogen bonding and π-π stacking, which restricts the rotational degrees of freedom of Cur and shields it from hydrolytic and oxidative attacks [64]. Moreover, Ant may function as a sacrificial UV absorber and antioxidant, preferentially neutralizing environmental stressors to preserve the biological activity of the encapsulated Cur.

### 3.2 Self-Assembly Mechanism of Cur-ant NPs

To explore the self-assembly mechanism of Cur-Ant NPs and verify the stoichiometric consistency of the binary system, we conducted a series of structural studies. The successful co-assembly of Cur and Ant was confirmed by UV-visible spectroscopy (Fig. 1g). The spectra of Cur-Ant NPs, particularly the optimized Cur-Ant (1:3) formulation, exhibited characteristic absorption peaks of Cur (shifted to ∼410 nm) and Ant (approx. 262 nm), confirming the robust integration of both polyphenols. Notably, the peak intensity ratios in the Cur-Ant (1:3) spectrum remained highly consistent with the initial feeding stoichiometry, suggesting minimal preferential loss of either component during the nanoprecipitation and subsequent dialysis processes. The observed blue shift in the Cur peak (from 430 nm to 410 nm) further signifies strong π-π stacking and H-aggregation between the transition dipoles of the two molecules [42]. This intense intermolecular affinity acts as a ‘molecular lock’ that stabilizes the binary complex, ensuring that the final nanoparticles maintain the targeted 1:3 M ratio. Such synchronized co-precipitation is a hallmark of carrier-free systems governed by high-affinity non-covalent interactions, which effectively prevents the deviation of the component ratio even under purification conditions.

Furthermore, fluorescence spectroscopy (Fig. 1h) revealed a significant quenching of Ant's intrinsic fluorescence emission (peak around 560 nm) in the co-assembled nanoparticles compared to free Ant. This quenching phenomenon strongly suggests close proximity and effective intermolecular interactions, such as π−π stacking or potential energy transfer, between Cur and Ant molecules within the nanostructure, which are essential drivers for the self-assembly process [52,65]. Furthermore, compared to Cur alone, Cur-Ant NPs exhibited a pronounced blue shift in fluorescence properties (progressively shifting from 580 nm to 560 nm and further to 550 nm) with increasing molar ratios of Ant. This phenomenon could be attributed to the self-assembly of Cur and Ant, which induces alterations in the energy level gaps between the excited and ground states.

FTIR spectroscopy (Fig. 1i) confirmed significant intermolecular interactions between Cur) and Ant upon their self-assembly into NPs. Compared to the pure compounds, the spectra of Cur-Ant NPs exhibited a noticeable shift and alteration in the broad -OH stretching band of Ant (originally ca. 3428.8 cm^−1^, shifting to ca. 3404.2-3381.1 cm^−1^ in NPs), alongside a distinct attenuation and/or shift in the characteristic C=O stretching vibration of Cur (originally ca. 1628.1 cm^−1^, shifting to ca. 1627.1-1626.7 cm^−1^ in NPs). These spectral changes strongly indicate the formation of intermolecular hydrogen bonds between the hydroxyl groups of Ant and the carbonyl moieties of Cur as a primary driving force for nanoparticle formation [66]. Additionally, subtle modifications in the aromatic C=C vibration regions (e.g., Cur's peak at 1603.5 cm^−1^, Ant's peak at 1624.7 cm^−1^, NP's peak at 1588.1-1587.1 cm^−1^) suggest potential contributions from π-π stacking interactions. Collectively, these observations substantiate the successful encapsulation of both molecules within a structurally integrated nano-assembly stabilized by these specific non-covalent interactions [43,46].

Powder X-ray diffraction (PXRD) was utilized to probe the physical state and structural alterations upon the self-assembly of Cur and Ant into nanoparticles (Cur-Ant NPs). As illustrated in Fig. 1j, pristine Cur exhibited a series of sharp, intense diffraction peaks across the 2θ range of 8-30°, unequivocally confirming its highly crystalline nature. In contrast, pure Ant presented a broad, diffuse halo centered at approximately 2θ = 23.2° (corresponding to an approximate d-spacing of 3.83 Å), characteristic of an amorphous or poorly crystalline material. Significantly, upon co-assembly into Cur-Ant NPs, whether at a 1:1 or 1:3 (Cur:Ant) molar ratio, the distinctive crystalline diffraction signature of Cur was virtually obliterated. Instead, the PXRD patterns of both Cur-Ant (1:1) and Cur-Ant (1:3) NPs were dominated by broad, featureless humps. Specifically, the Cur-Ant (1:1) NPs displayed a broad peak centered at 2θ ≈ 26.1° (d-spacing ≈ 3.41 Å), while the Cur-Ant (1:3) NPs showed a similar feature around 2θ ≈ 24.5° (d-spacing ≈ 3.63 Å). This disappearance of Cur's characteristic peaks, coupled with the emergence of these specific halos, indicates a profound molecular rearrangement and a transition from long-range crystalline order to a controlled, short-range ordered state [42]. The observed d-spacing values (∼3.4 - 3.6 Å) are highly characteristic of aromatic π-π stacking distances, suggesting that Cur and Ant molecules spontaneously organize into an ‘ordered’ supramolecular framework driven by directed intermolecular forces [43,67]. This amorphization is not a sign of random aggregation but a hallmark of successful, energetically favorable self-assembly that disrupts the native crystallinity of Cur to form a distinct, stable nanostructured entity with improved solubility and bioactivity [52,68].

UHPLC Q-Exactive Orbitrap HRMS (UHPLC-Q-HRMS) was utilized to analyze the constituent units of the Cur-Ant NPs. In the positive ion ESI mode, a predominant peak corresponding to a non-covalent complex formed between Cur and Ant ([Cur + Ant + H]^+^, calculated *m*/*z* 614.4939) was observed. Furthermore, ion peaks representing the individual components, namely curcumin ([Cur + H]^+^, calculated *m*/*z* 369.1282) and anthocyanin ([Ant]^+^, calculated *m*/*z* 287.0550), were also clearly identified in the spectrum (Fig. S1g). These results provide compelling evidence that the Cur-Ant NPs were successfully formed through the non-covalent self-assembly of curcumin and anthocyanin [42].

The molecular-level interactions and driving forces governing the formation of Cur-Ant NPs were unequivocally confirmed through a comparative ^1^H NMR analysis (Fig. 1k). Upon nano-assembly, the Cur-Ant NP spectrum exhibited profound alterations in chemical shifts ($\delta$) and signal morphology, providing critical insights into the supramolecular architecture. Specifically, the most dramatic perturbation regarding the hydroxyl groups was the disappearance of the phenolic hydroxyl proton of free Cur (originally at 9.64 ppm) and the emergence of a sharp singlet at 12.59 ppm, assigned to the stabilized enolic-OH. This transition, accompanied by the concentration of anthocyanin aromatic hydroxyl signals at 7.53 ppm, signifies that the phenolic protons of Cur become extensively involved as donors in a robust intermolecular hydrogen-bonding network with the hydroxyl-rich framework of Ant, while the nanoparticle environment rigidifies and stabilizes the enol tautomer of Cur.

Complementing the H-bonding network, the aromatic and olefinic scaffold of Cur demonstrated systematic and significant upfield (shielding) perturbations, which serve as a definitive spectroscopic signature of intense π-π stacking. Specifically, the aromatic protons H-13 (resonating at ≈ 7.56 ppm in free Cur) and H-7 (originally at 7.52 ppm) both shifted significantly upfield to 6.85 ppm (H-13′, Δδ = −0.71 ppm) and 6.83 ppm (H-7′, Δδ = −0.69 ppm), respectively, in the Cur-Ant NPs. Similar upfield perturbations were recorded for the aromatic protons H-19 and H-2 (shifting from ≈7.32 ppm to ≈ 6.38 ppm, Δδ = −0.94 ppm) and the protons H-6 and H-15 (shifting from ≈7.15 ppm to ≈ 6.19 ppm, Δδ = −0.96 ppm). These widespread shielding effects indicate that the aromatic rings of curcumin are deeply intercalated or stacked against the flavylium nuclei of anthocyanin, thereby experiencing the intense diamagnetic ring current of the Ant framework [69,70].

The degree of molecular proximity within the hydrophobic core was further highlighted by the perturbations of the central heptadienone chain and methoxy groups. The protons H-3 and H-18 (originally at ≈ 6.81–6.83 ppm) shifted significantly to ≈ 5.33–5.35 ppm (Δδ = −1.48 ppm), while the adjacent protons H-8 and H-12 (observed at ≈ 6.72–6.77 ppm) migrated to ≈ 5.27 ppm (Δδ ≈ −1.45 ppm). Most notably, the enolic methine proton (H-10) exhibited a massive upfield shift from 6.05 ppm to 4.38 ppm (Δδ = −1.67 ppm), where it exhibited a distinct signal overlap with the shifted Ant Sugar-H (α) proton (originally at ≈ 5.0 ppm). Simultaneously, the Ant signal originally at 5.5 ppm moved to a sharp independent peak at 5.00 ppm, and the Cur methoxy protons (H-5, H-16) shifted from 3.83 ppm to 3.38 ppm to merge with the various sugar-moiety protons of Ant (3.26–3.69 ppm). This synergistic shielding and widespread signal overlap suggest a co-assembly mechanism where the hydrophobic collapse driven by π-π stacking is reinforced by an extensive H-bonding network, resulting in a compact and stable carrier-free nanodelivery system [71].

### 3.3 Self-assembly molecular dynamics simulation of Cur-Ant NPs

To elucidate the self-assembly mechanism and intermolecular interactions between Ant and Cur at different stoichiometric ratios, molecular dynamics (MD) simulations were performed. We investigated two systems: a 1:1 M ratio of Ant:Cur and a 1:3 M ratio of Ant:Cur.

**Fig. 2:**
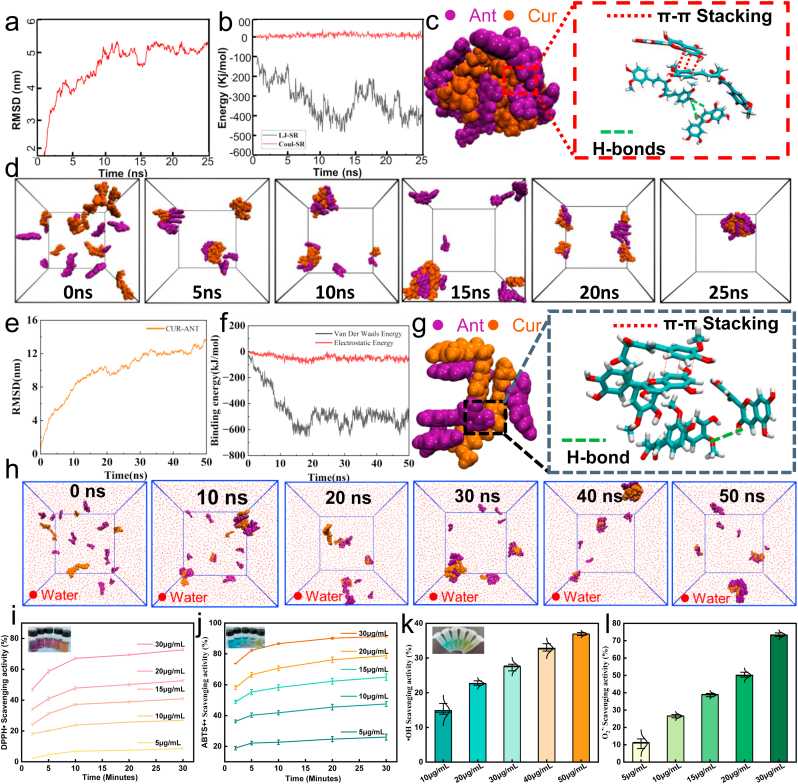
**Molecular Dynamics (MD) Simulation of Self-Assembly and *In Vitro* Antioxidant Activity of Cur-Ant NPs.** (a-d) MD simulation of the self-assembly process for the Cur-Ant (1:1) system over 25 ns: (a) Root mean square deviation (RMSD) of the complex over time. (b) Non-bonded interaction energies (Lennard-Jones and Coulombic) during the simulation. (c) Representative final aggregated structure, showing key intermolecular interactions (π-π stacking and H-bonds). (d) Snapshots of the simulation trajectory at different time points, visualizing the aggregation process. (e-h) MD simulation of the self-assembly process for the Cur-Ant (1:3) system over 50 ns: (e) RMSD of the complex over time. (f) Non-bonded interaction energies during the simulation. (g) Representative final aggregated structure, with inset showing intermolecular interactions. (h) Snapshots of the simulation trajectory at different time points. (i) DPPH radical scavenging activity of Cur-Ant NPs at different concentrations over time. (j) ABTS radical scavenging activity of Cur-Ant NPs at different concentrations over time. (k) Hydroxyl (•OH) radical scavenging activity of Cur-Ant NPs at different concentrations.

The structural stability and conformational changes of the Ant:Cur (1:1) system were monitored over a 25 ns simulation period. The root mean square deviation (RMSD) of the complex was calculated to assess the equilibration of the system. As depicted in Fig. 2a, the RMSD value gradually increased from the initial state, reaching a relatively stable plateau of approximately 5.0-5.5 nm after around 15 ns, indicating that the system achieved a stable conformational state. Representative snapshots of the simulation trajectory (Fig. 2d), taken at 5 ns intervals from 0 ns to 25 ns, visually illustrate the progressive aggregation of Ant and Cur molecules from an initially dispersed state into a distinct nanocluster. The solvent-accessible surface area (SASA) was analyzed to understand the changes in the exposure of the complex to the solvent (Fig. S2a). The SASA value exhibited a rapid decrease from an initial value of approximately 80 nm^2^ within the first 5 ns, eventually stabilizing in the range of 35-50 nm^2^. This reduction in SASA signifies the formation of more compact and stable nanoclusters, driven by the tendency to minimize exposure to the aqueous environment. The driving forces behind the self-assembly process were investigated by calculating the non-bonded interaction energies, specifically the short-range Lennard-Jones (LJ-SR) potential, representing van der Waals forces and π-π stacking interactions, and the short-range Coulombic (Coul-SR) potential, reflecting electrostatic interactions. As shown in Fig. 2b, the LJ-SR energy stabilized at a significantly more negative value (around −300 to −400 kJ/mol) compared to the Coul-SR energy (around −50 to −150 kJ/mol). This suggests that π-π stacking interactions and van der Waals forces are the dominant contributors to the stabilization of the Ant:Cur (1:1) nanoclusters, although electrostatic interactions also play a role. The formation of intermolecular hydrogen bonds was also monitored (Fig. S2b). The number of hydrogen bonds fluctuated throughout the simulation, generally ranging from 0 to 4, with an average of approximately 1-2, further contributing to the stability of the assembled structure. The final aggregated structure (Figure c) visually confirms the presence of both π-π stacking between the aromatic moieties of Ant and Cur and intermolecular hydrogen bonds.

To investigate the effect of stoichiometry on the self-assembly process, MD simulations were also conducted for an Ant:Cur system with a 1:3 M ratio over a 50 ns period. The RMSD profile for this system (Fig. 2e, labeled Cur-Ant, assuming Ant is in excess) showed a gradual increase, reaching a plateau around 10-12 nm after approximately 30-40 ns. This indicates that the 1:3 system also forms stable aggregates, though it equilibrates over a longer timescale and exhibits a larger RMSD value compared to the 1:1 system, potentially suggesting the formation of larger or more dynamic assemblies. Snapshots from the simulation (Fig. 2h, at 10 ns intervals from 0 ns to 50 ns) demonstrate the aggregation process, culminating in the formation of nanoclusters in the presence of water molecules. The SASA for the 1:3 Cur:Ant system (Fig. S2c) started at a higher value of approximately 110 nm^2^ and decreased to a stable range of 60-70 nm^2^ after about 20 ns? While a significant reduction, the final SASA is larger than that observed for the 1:1 system, which might be attributed to the formation of less compact nanoclusters due to the higher proportion of Ant. The binding energies for the 1:3 system, represented by Van der Waals energy and Electrostatic energy (Fig. 2f), showed that Van der Waals interactions were the primary driving force, stabilizing around −500 to −650 kJ/mol. Electrostatic energies were less negative, fluctuating around −50 to −100 kJ/mol. The magnitude of Van der Waals energy is notably more negative than in the 1:1 system, possibly due to the increased number of Ant molecules available for π-π stacking and other Van der Waals interactions. The number of hydrogen bonds in the 1:3 system (Fig. S2d) ranged from 0 to approximately 3, with an average count similar to the 1:1 system (around 1-1.5), indicating their persistent role in mediating intermolecular connections. The final structure (Fig. 2g) illustrates these π-π stacking and hydrogen bonding interactions within the 1:3 Cur:Ant aggregates.

Molecular dynamics simulations reveal that both 1:1 and 1:3 M ratios of Cur:Ant readily self-assemble into stable nanoclusters in an aqueous environment [42]. The primary driving forces for this assembly are π-π stacking interactions and van der Waals forces, with contributions from hydrogen bonding and electrostatic interactions. The 1:1 Cur:Ant system reached equilibrium more rapidly and formed more compact nanoclusters, as suggested by the lower RMSD and SASA values. In contrast, the 1:3 Cur:Ant system took longer to equilibrate and resulted in structures with higher RMSD and SASA values, possibly indicative of larger or more extended aggregates. The increased proportion of Anthocyanin in the 1:3 system led to stronger overall Van der Waals interaction energies, highlighting the significant role of Ant in mediating these attractive forces [72]. In both systems, π-π stacking interactions, facilitated by the aromatic ring structures of both Ant and Cur, appear to be the predominant stabilizing force, significantly outweighing the energetic contributions from direct Coulombic interactions and the specific hydrogen bonds quantified. However, hydrogen bonds are consistently present and contribute to the specificity and stability of the intermolecular network. These findings underscore the complex interplay of non-covalent interactions governing the self-assembly of Ant and Cur, with the molar ratio influencing the dynamics, timescale, and structural characteristics of the resulting nano-architectures. This detailed understanding of the assembly process and the involved interactions is crucial for designing and optimizing Ant-Cur based delivery systems or functional materials.

### 3.4 Radical scavenging ability of Cur-Ant NPs

The ability of antioxidants to donate hydrogen atoms or electrons to neutralize free radicals is a key mechanism of their action. The DPPH• and ABTS•+ assays are widely employed to evaluate this capacity, even in complex systems [73]. The strong UV-vis absorbance peak of DPPH• at 517 nm was greatly reduced when different concentrations of Cur-Ant NPs were added, and the color of the solution changed from purple to yellow (Fig. 2i), indicating that free radicals were gradually scavenged. At a concentration of 30 μg/mL, the NPs achieved approximately 75% scavenging of DPPH• radicals after 30 min of incubation. Lower concentrations also exhibited considerable activity, for example, 20 μg/mL of NPs scavenged over 50% of DPPH• radicals within the same timeframe. ABTS can react with an oxidizing agent to produce stable free radicals of ABTS+• and the solution appears blue. Similarly, when different concentrations of Cur-Ant NPs were added, UV− vis absorbance was decreased in a concentration-dependent manner at 734 nm and the solution was decolorized (Fig. 2j). It is noteworthy that about 90% of free radicals of ABTS+• could be removed in 10 min when the final concentration of Cur-Ant NPs was 30 μg/mL. Interestingly, the scavenging kinetics of Cur-Ant NPs for both stable (DPPH•) and transient (ABTS•+) free radicals show a rapid initial phase, followed by a more gradual increase, approaching a plateau by 30 min. In addition, we also measured the ability of free Cur and Ant to scavenge DPPH• and ABTS+•, and found that the scavenging ability of NPs was mainly attributed to Cur (Fig. S2e and S2f).

Oxidative stress is characterized by an overproduction of reactive oxygen species (ROS), which can overwhelm endogenous antioxidant defenses and lead to cellular damage to lipids, proteins, and DNA [74]. Among these, the hydroxyl radical (•OH) is regarded as one of the most reactive and detrimental ROS in biological systems. We therefore assessed the •OH scavenging potential of the Cur-Ant NPs. The test substance exhibited potent and dose-dependent scavenging activity against hydroxyl radicals (•OH), as shown in Fig. 2k. The characteristic blue color of TMB, resulting from its oxidation by •OH, was progressively inhibited by the addition of the substance (10, 20, 30, 40 and 60 μg/mL). This visual attenuation correlated directly with the measured •OH clearance rate, which reached a maximum of approximately 37% at the 50 μg/mL concentration. Given that the superoxide anion radical (O_2_^•-^) is a key primary ROS and acts as a precursor to more aggressive species like •OH and H_2_O_2_, we further evaluated the O_2_^•-^ scavenging capability of Cur-Ant NPs. As illustrated in Fig. 2l, the NPs exhibited a robust and concentration-dependent capacity to quench O_2_^•-^ radicals. Specifically, Cur-Ant NPs achieved average scavenging efficiencies of 10.96%, 26.58%, 38.85%, and 50.16% at concentrations of 5, 10, 15, and 20 μg/mL, respectively. Remarkably, the scavenging rate escalated to 73.33% at a concentration of 30 μg/mL, highlighting the exceptional efficacy of the nanoparticles in neutralizing this fundamental radical within the biological oxidative cascade. In summary, the Cur-Ant self-assembled nanoparticles demonstrate potent and versatile antioxidant activities by effectively scavenging DPPH•, ABTS•+, and biologically significant ROS, including •OH and O_2_^•-^ radicals. DPPH•, ABTS•+, and highly reactive •OH radicals.

## 4 Cur-Ant NPs attenuate DSS-Induced Colitis in Zebrafish model

### 4.1 Biocompatibility profile and systemic therapeutic efficacy

To ensure the clinical translational potential of the formulation, we first evaluated the in vivo biocompatibility using zebrafish embryos and larvae. The zebrafish model was strategically selected as a high-throughput vertebrate bridge between in vitro cellular assays and murine studies, offering unique advantages such as optical transparency for real-time toxicity monitoring and high physiological conservation of the intestinal barrier and innate immune responses with mammals [75,76]. As shown in Fig. S3a/b, the safety profile of all treatment groups was assessed across a concentration gradient. Contrary to the potential cytotoxicity often associated with chemotherapeutic agents, neither the Cur-Ant NPs nor the free monomeric components (Cur and Ant) induced any significant adverse effects on embryo hatchability or larval survival rates at the tested concentrations. The survival outcomes in all treated groups were statistically indistinguishable from the untreated control, confirming that Cur-Ant NPs possess excellent developmental and systemic biocompatibility. This first-tier in vivo validation reinforces the safety credentials of the synthesized Cur-Ant NPs and provides a robust foundation for evaluating their therapeutic efficacy in a complex physiological environment prior to mammalian trials.

**Fig. 3:**
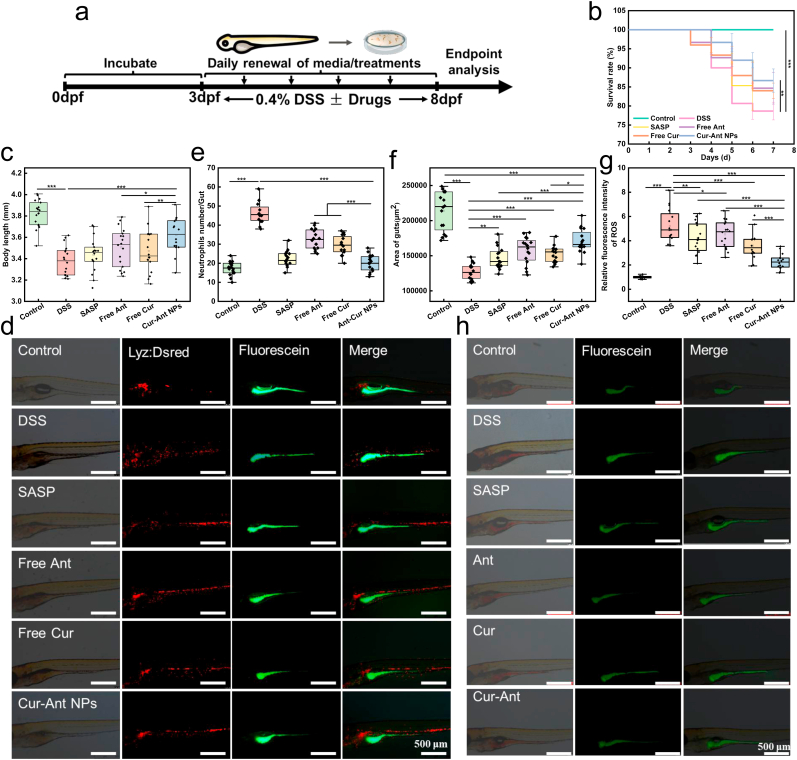
**Cur-Ant NPs Ameliorate DSS-Induced Colitis in Zebrafish by Inhibiting Neutrophil Infiltration and Oxidative Stress.** (a) Schematic illustration of the experimental protocol. (b) Survival rates of zebrafish larvae across different treatment groups monitored over 8 days. (c) Statistical analysis of the body length of zebrafish larvae at 8 dpf. (d) Representative fluorescence microscopy images of Tg (lyz:DsRed2) transgenic zebrafish larvae. Neutrophil migration in the intestine is shown in red (Lyz:DsRed channel), and intestinal morphology is visualized by fluorescein staining (green channel). Scale bars, 500 μm. (e) Quantification of the number of infiltrated neutrophils in the intestinal region. (f) Quantitative analysis of the intestinal area (μm^2^) calculated using ImageJ. (g) Representative fluorescence images showing reactive oxygen species (ROS) levels in the intestine of 8 dpf zebrafish larvae, detected by DCFH-DA staining (green). Scale bars, 500 μm. (h) Quantification of the fluorescence intensity of ROS in the intestine analyzed using ImageJ. Data in (c, e, f, h) are presented as box plots, and statistical significance was analyzed using one-way ANOVA (n = 15 larvae per group). ∗*p* < 0.05, ∗∗*p* < 0.01, ∗∗∗*p* < 0.001 vs the DSS group. (For interpretation of the references to color in this figure legend, the reader is referred to the Web version of this article.)

Following this safety confirmation, the therapeutic efficacy was evaluated in a DSS-induced IBD zebrafish model, established according to the experimental timeline illustrated in Fig. 3a. Zebrafish larvae (3 dpf) were exposed to 0.4% DSS with or without concomitant drug treatment until 8 dpf. The DSS challenge induced severe systemic toxicity, manifesting as a sharp decline in survival rate (Fig. 3b) and marked growth retardation (Fig. 3c). A pivotal aspect of our experimental design throughout these trials is the fixed-dose ratio strategy, which provides a stringent benchmark for evaluating the efficiency of the nanotechnology platform. Unlike conventional component-equivalent studies, all treatment groups were administered at the same total mass dosage. Under this framework, the Cur-Ant NPs—which contain only a fractional dose of Cur (≈25%) and Ant (≈75%)—were directly challenged against ‘full-strength’ (100% mass) doses of the individual free monomers. While therapeutic interventions with SASP and free drugs (Ant and Cur) offered partial relief, the Cur-Ant NPs delivered markedly superior therapeutic outcomes, maintaining a high survival rate comparable to the healthy control group (Fig. 3b). The fact that the nanoparticles significantly outperformed these higher-dose free drug benchmarks unequivocally demonstrates that their therapeutic superiority is fundamentally driven by the structural advantages of the carrier-free assembly. This nanostructural integration overcomes the inherent limitations of the free components, such as the poor aqueous solubility of Cur and the rapid degradation of Ant, by ensuring their spatial and temporal synchronization. As shown in Fig. 3c, DSS treatment caused a significant reduction in larval body length compared to the control group. Notably, treatment with Cur-Ant NPs effectively reversed this phenotype. The body length in the Cur-Ant NP-treated group was restored to levels statistically indistinguishable from the healthy controls (approx. 3.7–3.8 mm), an effect significantly superior to that of the free drug groups. These macroscopic findings underscore the advantage of the self-assembled nanoplatform in enhancing the bioavailability and therapeutic index of the bioactive compounds against colitis-induced systemic damage.

### 4.2 Restoration of intestinal barrier integrity and modulation of inflammatory microenvironment

**Fig. 4:**
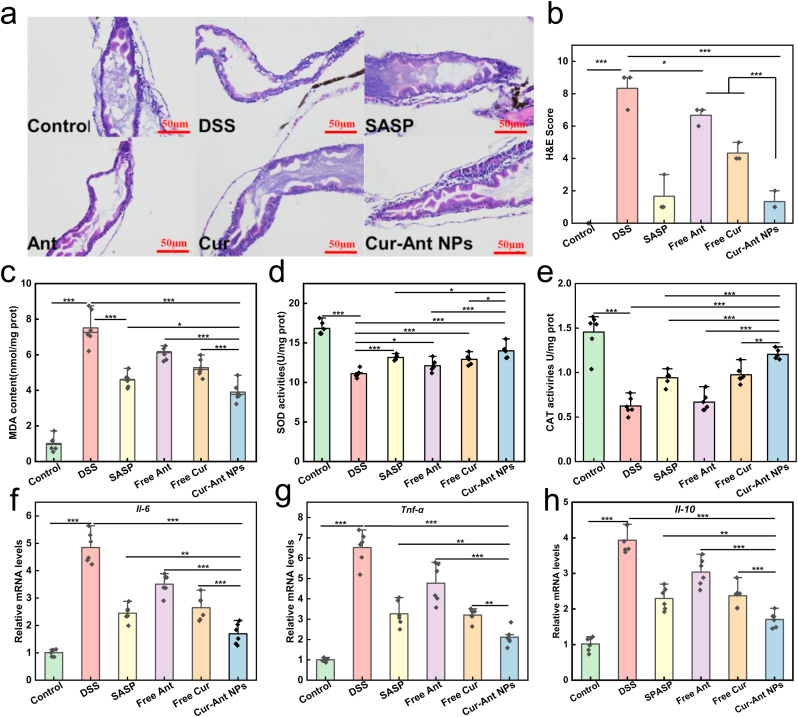
**Cur-Ant NPs Attenuate Oxidative Stress, Modulate Inflammatory Gene Expression, and Restore Intestinal Integrity in the Zebrafish Colitis Model.** (a–c) Effects of Cur-Ant NPs on oxidative stress markers in 8 dpf zebrafish larvae: (a) superoxide dismutase (SOD) activity, (b) catalase (CAT) activity, and (c) malondialdehyde (MDA) content. (d–f) Relative mRNA expression levels of inflammatory cytokines in zebrafish larvae determined by qRT-PCR: (d) *IL-6*, (e) *TNF-α*, and (f) *IL-10*. (g) Representative images of Hematoxylin and Eosin (H&E) stained mid-to-posterior intestinal sections from different treatment groups (Ant: Free Ant; Cur: Free Cur). Scale bars, 50 μm. (h) Histological scoring of intestinal damage based on H&E stained sections. Data are presented as mean ± SD. Sample sizes are n = 6 for oxidative stress analysis (a–c) and n = 3 for gene expression and histological scoring (d–f, h). Statistical significance was analyzed using one-way ANOVA. ∗*p* < 0.05, ∗∗*p* < 0.01, ∗∗∗*p* < 0.001.

To elucidate the mechanisms driving the systemic recovery, we integrated histological examination with biochemical and molecular analyses. At the tissue level, DSS induction severely compromised the intestinal barrier integrity. As visualized by the oral fluorescein angiography (Fig. 3d, green channel) and quantified in Fig. 3f, the DSS group exhibited a significant contraction in intestinal area due to severe mucosal damage [77]. This was corroborated by H&E staining (Fig. 4a), which revealed extensive architectural disruption, characterized by villous atrophy, epithelial vacuolation, and loss of goblet cells, corresponding to a high histological score (Fig. 4b). Concomitantly, a massive infiltration of neutrophils—a hallmark of acute inflammation—was observed in the gut region, indicated by the massive accumulation and diffuse infiltration of discrete *lyz:DsRed*-labeled neutrophils in the gut region (Fig. 3d, red channel), which was further validated by the quantitative analysis of individual cell counts (Fig. 3e). Treatment with Cur-Ant NPs exerted a profound restorative effect on the mucosal barrier. The nanoformulation effectively suppressed the transmigration of neutrophils (Fig. 3e) and reversed the intestinal atrophy, restoring the gut area to near-normal levels (Fig. 3f). Histologically, the Cur-Ant NP-treated group displayed a striking restoration of gut architecture, featuring tall, well-organized villous folds and a regenerated epithelium (Fig. 4a) [78], resulting in a significantly reduced histological score comparable to the SASP positive control (Fig. 4b).

Underpinning this structural restoration was the potent modulation of the oxidative and inflammatory microenvironment. The DSS-induced pathology was driven by a profound oxidative imbalance, manifested as a dramatic surge in intestinal ROS levels (Fig. 3g and h) [79]. This was accompanied by elevated lipid peroxidation (MDA content, Fig. 4c) and a depletion of antioxidant enzymes (SOD and CAT, Fig. 4d and e). Meanwhile, Cur-Ant NPs treatment acted as a robust antioxidant defense, quenching ROS overproduction and reinstating the activities of SOD and CAT, thereby protecting intestinal cells from oxidative damage. Furthermore, gene expression analysis (Fig. 4f–h) confirmed that the formulation resolved the cytokine storm. In the DSS group, key pro-inflammatory cytokines (*Il6* and *Tnf-α*) were significantly upregulated [79]. Interestingly, the expression of the anti-inflammatory cytokine *Il-10* was also markedly elevated compared to the control group (Fig. 4h), suggesting a compensatory physiological response attempting to counteract the severe inflammation [77]. The Cur-Ant NPs intervention proved effective in resetting this immune profile: it not only downregulated *Il-6* and *Tnf-α* but also normalized the elevated *Il-10* levels. This indicates that Cur-Ant NPs facilitate tissue repair by eliminating the root inflammatory stimuli, thereby reducing the physiological need for a compensatory anti-inflammatory response [80].

It is worth emphasizing that the markedly superior therapeutic efficacy of Cur-Ant NPs over their free monomeric counterparts is not a result of simple pharmacological addition, but rather a reflection of the unique structural and functional synergy conferred by the co-assembled nanoplatform. As demonstrated in similar binary carrier-free systems, such as berberine-magnolol or berberine-hesperetin, the transition from individual small molecules into a unified nanostructure facilitates a “1 + 1 > 2″ synergistic effect. In a simple physical mixture, the pronounced hydrophobicity of Cur would inevitably lead to heterogeneous phase separation and rapid sedimentation in the aqueous gastrointestinal environment, hindering its effective interaction with the mucosa. Conversely, the Cur - Ant NPs function as an integrated, stabilized “molecular cage” that ensures the synchronized delivery of both Cur and Ant to the inflammatory sites [42,43,46]. This co-assembly not only optimizes the solubility of Cur but also leverages the sub-micron particle size for targeted accumulation via the “ELVIS” effect, achieving a significantly higher local concentration of both antioxidants and anti-inflammatory agents at the lesion site compared to a simple additive combination.

### 4.3 Modulation of intestinal microbiota

Specific components of the intestinal microbiota have been persistently linked to pathogenesis of IBD [77]. To elucidate the impact of Cur-Ant NPs treatment on the intestinal ecosystem, we performed 16 S rRNA gene sequencing on the gut microbiota of zebrafish. As shown in Fig. S4c, principal coordinates analysis (PCoA) revealed a significant separation of the microbial community structure between the DSS-treated group and the Control group along the PCoA1 axis (accounting for 55.05% of the variance; PERMANOVA, P = 0.005), indicating that DSS induced pronounced gut dysbiosis [79]. Importantly, treatment with Cur-Ant NPs initiated a compositional shift away from the dysbiotic state established by DSS, effectively remodeling the gut microbial landscape. Furthermore, Analysis of species richness (Observed OTUs) (Fig. S4a) and diversity (Shannon index) (Fig. 4b) revealed no statistically significant differences among the Control, DSS, and Cur-Ant NPs groups (P > 0.05 for all comparisons). To identify the key taxa driving the community-level shifts observed in our PCoA analysis despite this stability in alpha diversity, we examined the relative taxonomic abundances. At the taxonomic level (Fig. S4d), the DSS-induced dysbiosis was characterized by a dramatic expansion of the phylum Proteobacteria, a well-established hallmark of intestinal inflammation [81]. This was accompanied by a sharp increase in the relative abundance of opportunistic pathogenic genera, such as *Stenotrophomonas*. The administration of Cur-Ant NPs decisively counteracted these changes. It significantly suppressed the bloom of *Proteobacteria* and *Bacteroidetes*, and concurrently fostered the growth of other phyla, including *Actinobacteria* and *Firmicutes*. At the genus level (Fig. S4e), the nanoformulation obviously reduced the abundance of *Stenotrophomonas* and *Runella*, while promoting the recovery of diverse commensal bacteria, such as *Mycobacterium* and *Aeromonas*. Taken together, these findings indicate that the therapeutic efficacy of Cur-Ant NPs in this IBD model is strongly associated with their ability to remodel the gut microbiota. By suppressing opportunistic pathogens and promoting a diverse, balanced community, the nanoformulation effectively ameliorates gut dysbiosis, which is a key mechanism underlying its potent anti-inflammatory effects.

## 5 Therapeutic efficacy of Cur-Ant NPs in DSS-induced IBD mice model

### 5.1 In Vivo Biodistribution and Biosafety Evaluation

**Fig. 5:**
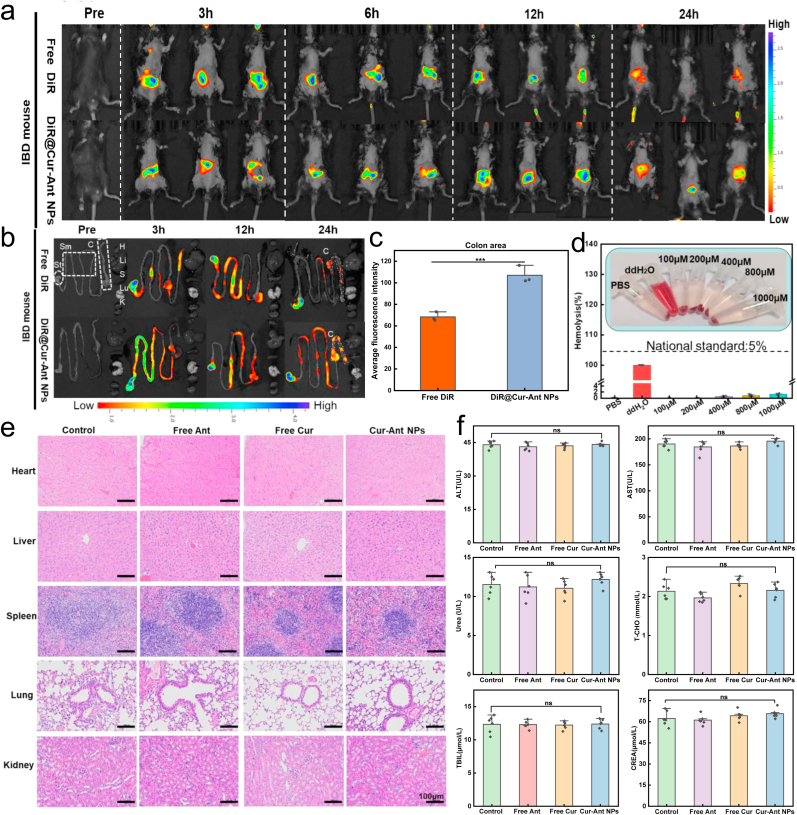
**In Vivo Biodistribution and Biosafety Evaluation of Cur-Ant NPs.** (a) In vivo fluorescence imaging of IBD mice at different time points (Pre, 3, 6, 12, and 24 h) after oral administration of Free DiR and DiR-labeled Cur-Ant NPs (DiR@Cur-Ant NPs). (b) Ex vivo fluorescence images of the gastrointestinal tract harvested from mice at indicated time points, displaying the retention of nanoparticles. (c) Quantitative analysis of the average fluorescence intensity in the colon area at 24 h. (d) Hemolysis assay of mouse red blood cells after incubation with different concentrations of Cur-Ant NPs. The dashed line indicates the national safety standard of 5%. Inset: photographs of the corresponding samples after centrifugation. (e) Representative Hematoxylin and Eosin (H&E) stained images of major organs (heart, liver, spleen, lung, and kidney) from mice after 13 days of treatment with free Ant, free Cur, or Cur-Ant NPs. Scale bars, 100 μm. (f) Serum biochemical analysis of key indicators for liver and kidney function, including Alanine Aminotransferase (ALT), Aspartate Aminotransferase (AST), Urea, Total Cholesterol (T-CHO), Total Bilirubin (TBIL), and Creatinine (CREA). Data in (c, d, f) are presented as mean ± SD (n = 3 for fluorescence quantification; n = 6 for biosafety assays). ∗∗∗*p* < 0.001. NS indicates no significant difference compared to the Control group. (For interpretation of the references to color in this figure legend, the reader is referred to the Web version of this article.)

Effective accumulation and prolonged retention of therapeutic agents in the inflamed colon are prerequisites for successful IBD treatment, as they maximize local drug concentration while minimizing systemic exposure. To investigate the gastrointestinal transit behavior and colonic targeting capability of the nanoplatform, the near-infrared lipophilic fluorescent dye DiR was encapsulated within the Cur-Ant NPs (DiR@Cur-Ant NPs) to serve as a tracer. The in vivo biodistribution was monitored using an IVIS spectrum imaging system following oral administration to mice. As shown in Fig. 5a, the fluorescence signal in the mice treated with free DiR was relatively weak and moved rapidly through the gastrointestinal tract, indicating rapid clearance. In stark contrast, the DiR@Cur-Ant NPs group exhibited a significantly stronger fluorescence intensity in the abdominal region, which persisted for up to 24 h. To precisely locate the distribution of the nanoparticles, the gastrointestinal tracts were harvested and imaged ex vivo (Fig. 5b). The results corroborated the in vivo findings: while the fluorescence of free DiR was negligible in the colon at 24 h, the DiR@Cur-Ant NPs showed robust accumulation specifically in the colonic region. Quantitative analysis (Fig. 5c) further confirmed that the average fluorescence intensity in the colon area of the NP-treated group was significantly higher than that of the free DiR group (*p* < 0.001). This enhanced colonic retention is likely attributed to the appropriate particle size and surface properties of the self-assembled nanoparticles, especially their strongly negative zeta potential (−39 mV). While electrostatic repulsion between the nanoparticles and the negatively charged mucus layer is theoretically present, the abundant phenolic hydroxyl groups on the NP surface—derived from the high proportion of Anthocyanin—facilitate multi-valent hydrogen bonding and hydrophobic interactions with the oligosaccharide chains of mucin glycoproteins. This mucoadhesive energy effectively overcomes the electrostatic repulsion barrier, ensuring prolonged retention at the inflamed lesions [56]. Furthermore, the high negative surface charge is crucial for maintaining colloidal stability and preventing self-aggregation, allowing the nanoparticles to effectively penetrate and interact with the complex colonic microenvironment. These unique surface properties, combining stability-promoting charge with adhesion-promoting hydrogen bonding, ensure a sustained therapeutic window at the disease site [58,82].

To evaluate whether the oral Cur-Ant NPs are suitable for use in humans, an extensive assessment of their biocompatibility and systemic safety must be performed. Hence, we conducted a battery of assays on both in vitro and in vivo systems. The hemocompatibility of the nanoformulation, a critical parameter for materials intended for biological application, was first assessed via a hemolysis assay. As depicted in Fig. 5d, the Cur-Ant NPs exhibited negligible hemolytic activity across a wide range of concentrations (100–1000 μM). Even at the highest tested concentration, the hemolysis rate remained well below the internationally accepted safety threshold of 5%, demonstrating the excellent blood compatibility of the nanoparticles. Furthermore, the in vivo systemic safety was investigated in healthy mice following oral administration. Histological analysis via H&E staining of major organs (including the heart, liver, spleen, lung, and kidney), revealed no discernible pathological abnormalities, lesions, or inflammatory cell infiltration in the Cur-Ant NP-treated group when compared to the healthy control group (Fig. 5e). More importantly, to further quantify potential organ toxicity, key serum biochemical indicators for liver and kidney function were measured. The levels of alanine aminotransferase (ALT), aspartate aminotransferase (AST), total bilirubin (TBIL), creatinine (CREA), total cholesterol (T-CHO), and urea in the Cur-Ant NP group showed no significant deviations from those of the healthy control group (Fig. 5f). This lack of change in critical biomarkers provides strong evidence that the Cur-Ant NPs do not induce hepatotoxicity or nephrotoxicity. Taken together, the above three types of tests provide convincing evidence indicating that Cur-Ant NPs possess superior biocompatibility and low systemic toxicity.

### 5.2 Amelioration of clinical symptoms, inflammatory cytokines, and histological damage

**Fig. 6:**
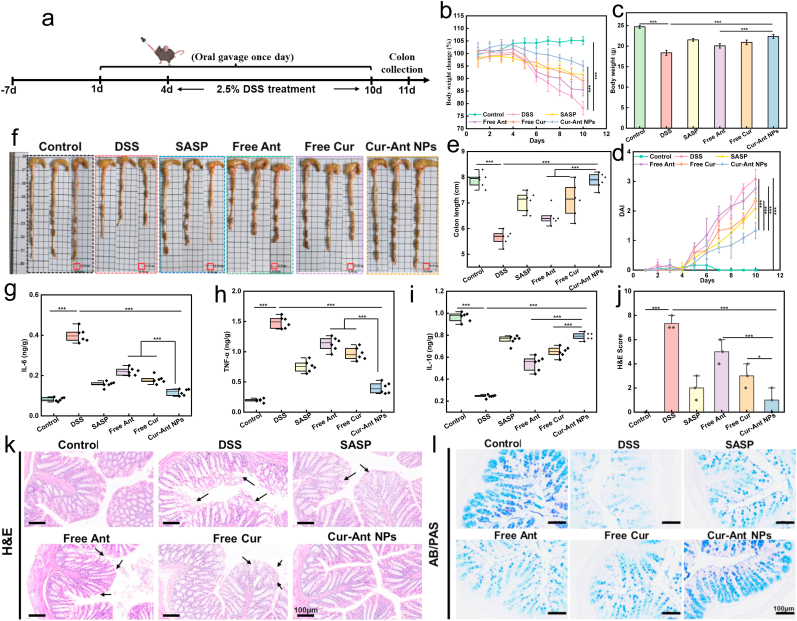
**Oral Administration of Cur-Ant NPs Alleviates Clinical Symptoms and Modulates Inflammatory Response in DSS-Induced Colitis Mice.** (a) Schematic illustration of the experimental design and treatment regimen. (b–f) Assessment of clinical signs of colitis: (b) body weight changes monitored daily, (c) final body weights at day 10, (d) Disease Activity Index (DAI) scores, (e) colon length statistics, and (f) representative macroscopic photographs of colons from each group. (g–i) Levels of inflammatory cytokines in colonic tissues determined by ELISA: (g) IL-6, (h) TNF-α, and (i) IL-10. (j) Histological scoring of colonic damage. (k) Representative Hematoxylin and Eosin (H&E) stained images of colon sections showing mucosal architecture. Scale bars, 100 μm. (l) Representative Alcian Blue/Periodic Acid-Schiff (AB/PAS) staining images indicating mucus secretion and goblet cells. Scale bars, 100 μm. Data are presented as mean ± SD. Sample sizes are n = 8 for clinical metrics (b–e), n = 6 for cytokine analysis (g–i), and n = 3 for histological scoring (j). Statistical significance was determined by one-way ANOVA. ∗*p* < 0.05, ∗∗*p* < 0.01, ∗∗∗*p* < 0.001. (For interpretation of the references to color in this figure legend, the reader is referred to the Web version of this article.)

Building on their promising effects in the zebrafish model, the in vivo therapeutic efficacy of Cur-Ant NPs was further validated using a well-established DSS-induced colitis mouse model (Fig. 6a). The successful establishment of acute colitis was confirmed after 7 days of DSS administration. Compared to the healthy control group, mice in the DSS model group exhibited a rapid and significant loss of body weight (Fig. 6b, c), a progressively increasing Disease Activity Index (DAI) score indicative of severe diarrhea and rectal bleeding (Fig. 6d), and a dramatic shortening of the colon (Fig. 6e, f), a cardinal sign of intestinal inflammation [82]. The therapeutic intervention with Cur-Ant NPs demonstrated exceptional efficacy in alleviating these clinical manifestations. Mice receiving Cur-Ant NPs via oral gavage exhibited significant protection against DSS-induced body weight loss, surpassing the therapeutic effects of the individual free compounds and the clinical reference drug, SASP (Fig. 6b and c). This improvement was further reflected by a marked decrease in the DAI (Fig. 6d) and the near-complete prevention of colon shortening, with colon lengths restored to levels statistically comparable to the healthy control group (Fig. 6e and f).

To elucidate the immunomodulatory mechanism, the expression of key inflammatory cytokines in colon tissue was quantified. The DSS group exhibited a massive upregulation of pro-inflammatory mediators TNF-α and IL-6 (Fig. 6g and h). In striking contrast, the expression of IL-10 (Fig. 6i), an essential anti-inflammatory cytokine, was significantly depleted. Treatment with the individual free compounds, Free Ant or Free Cur, provided only partial mitigation of this cytokine imbalance. Consistent with its potent anti-inflammatory role, Free Cur generally demonstrated superior efficacy in suppressing TNF-α and IL-6 compared to Free Ant, yet neither free drug could fully normalize the inflammatory profile. However, the Cur-Ant NPs treatment exhibited a remarkably enhanced therapeutic effect, powerfully rebalancing this cytokine profile by suppressing TNF-α and IL-6 to near-normal levels while robustly restoring IL-10 expression, significantly outperforming both free monomeric components.

Furthermore, we assessed the protective effects on colonic tissue architecture. H&E staining (Fig. 6k) revealed that DSS induction caused extensive crypt destruction, loss of glandular structures, and massive inflammatory cell infiltration, corresponding to a high histological score (Fig. 6j). While the groups treated with Free Ant and Free Cur showed moderate alleviation of tissue damage, considerable inflammatory infiltration and architectural disruption persisted. In marked contrast, Cur-Ant NPs conferred profound protection, synergistically preserving the glandular architecture and reducing inflammatory infiltration more effectively than either individual drug. Additionally, AB/PAS staining (Fig. 6l) indicated a severe depletion of the protective mucus layer in the DSS group. Although free drugs offered some protection, Cur-Ant NP treatment prompted a robust restoration of the goblet cell population and mucus secretion, far exceeding the effects of free compounds or SASP. Collectively, these results demonstrate that the nano-assemblies of Cur and Ant exert a powerful synergistic effect, effectively ameliorating colitis by mitigating clinical symptoms, resolving cytokine imbalance, and preserving intestinal tissue integrity.

### 5.3 Restoration of intestinal barrier function and inhibition of oxidative stress

**Fig. 7:**
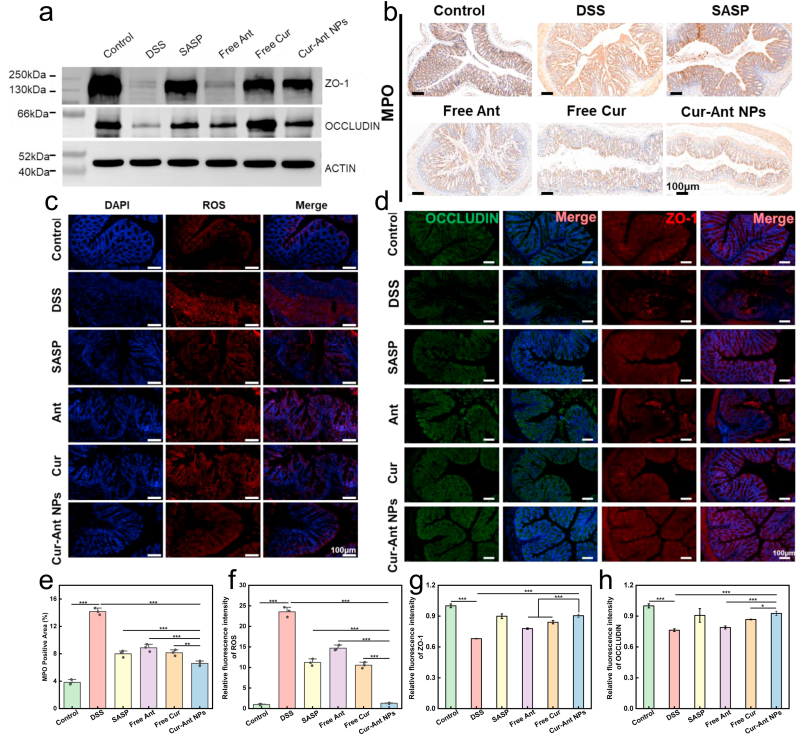
**Cur-Ant NPs Restore Intestinal Barrier Integrity and Suppress Neutrophil Infiltration and Oxidative Stress.** (a) Western blot analysis of tight junction proteins (ZO-1 and Occludin) in colonic tissues. Actin was used as a loading control. (b) Representative immunohistochemical (IHC) staining images of Myeloperoxidase (MPO) in colon tissues, indicating neutrophil infiltration. Scale bars, 100 μm. (c) Representative fluorescence images of reactive oxygen species (ROS) in colon tissues. ROS signal is shown in red, and nuclei are stained with DAPI (blue). Scale bars, 100 μm. (d) Representative immunofluorescence images showing the expression and localization of tight junction proteins. Occludin is stained green, ZO-1 is stained red, and nuclei are counterstained with DAPI (blue). Scale bars, 100 μm. (e) Quantification of MPO-positive areas based on IHC staining. (f) Quantification of the relative fluorescence intensity of ROS. (g, h) Quantitative analysis of the relative fluorescence intensity of (g) ZO-1 and (h) Occludin. Data in (e–h) were analyzed using ImageJ and are presented as mean ± SD (n = 3 independent fields/experiments). Statistical significance was determined by one-way ANOVA. ∗*p* < 0.05, ∗∗*p* < 0.01, ∗∗∗*p* < 0.001. (For interpretation of the references to color in this figure legend, the reader is referred to the Web version of this article.)

Given that the integrity of the intestinal barrier is critically dependent on intercellular tight junctions (TJs), we investigated the effect of Cur-Ant NPs on the key TJ proteins, ZO-1 and OCCLUDIN. Western blot analysis (Fig. 7a, S5a, S5b) confirmed that the protein expression levels of both ZO-1 and OCCLUDIN were significantly downregulated in the colons of the DSS group. While treatment with Free Ant and Free Cur provided a partial recovery of TJ expression, Cur-Ant NPs demonstrated a significantly more robust rescue effect, upregulating both proteins to levels comparable with the control group. This enhanced protection highlights the pharmacological synergy of the nano-integration: Ant likely mitigates the initial ROS-driven oxidation of junctional complexes, while Cur blocks the subsequent cytokine-mediated degradation of TJs. Consistent with these findings, immunofluorescence staining (Fig. 7d) showed that DSS treatment disrupted the continuous, honeycomb-like network of TJs. Treatment with Cur-Ant NPs dramatically reversed this damage, restoring the strong, continuous localization of both OCCLUDIN and ZO-1 at the cell boundaries (quantified in Fig. 7g and h), far surpassing the fragmented recovery observed in the free drug groups.

To further explore the mechanisms underlying this barrier protection, we evaluated the levels of oxidative stress and neutrophil infiltration in the colon tissues. ROS overproduction is a major driver of tissue damage in IBD. As shown in Fig. 7c, the DSS group exhibited intense ROS fluorescence signal in the colon. Notably, consistent with its superior radical-scavenging capacity derived from its unique catechol structure, Free Ant exhibited a stronger reduction in ROS than Free Cur. However, neither free monomer could achieve the profound quenching effect observed with the Cur-Ant NP treatment (Fig. 7f). Furthermore, Myeloperoxidase (MPO), a marker of neutrophil infiltration, was markedly elevated in the DSS group (Fig. 7b). In contrast to the ROS results, Free Cur was found to be more effective than Free Ant in reducing MPO positive areas, aligning with its potent role in suppressing downstream inflammatory recruitment. Crucially, Cur-Ant NPs demonstrated a powerful synergistic suppression of MPO (Fig. 7e), more effectively inhibiting neutrophil recruitment than either individual component. Collectively, these results demonstrate that the Cur-Ant NPs exert a comprehensive therapeutic effect through the synchronized delivery of its two components, reinforcing the physical barrier by restoring TJ proteins (ZO-1, OCCLUDIN) while simultaneously mitigating the damaging effects of oxidative stress and inflammatory infiltration.

The pronounced efficacy of Cur-Ant NPs in restoring mucosal integrity and alleviating oxidative stress underscores the unique advantages of structural integration over a simple physical mixture. Unlike a random combination where Cur and Ant exhibit divergent solubilities and disparate distribution patterns, the co-assembled nanostructure ensures the spatial synchronization of both agents within the same inflamed micro-niche. This integrated therapeutic plan prevents the asynchronous clearance of the monomers, allowing them to act simultaneously on the damaged epithelial cells to repair the tight junction network while concurrently neutralizing localized ROS. Such co-localized action facilitates a micro-domain normalization that a physical mixture—limited by the rapid phase-separation of Cur—cannot achieve. By locking the two polyphenols into a stable, nanoconfined state, the Cur-Ant NPs convert a simple pharmacological additive effect into a robust, structure-dependent synergy, as recently documented in other advanced carrier-free binary systems for inflammatory and barrier-related diseases [42,43].

### 5.4 Anti-inflammatory mechanism of Cur-Ant NPs

**Fig. 8:**
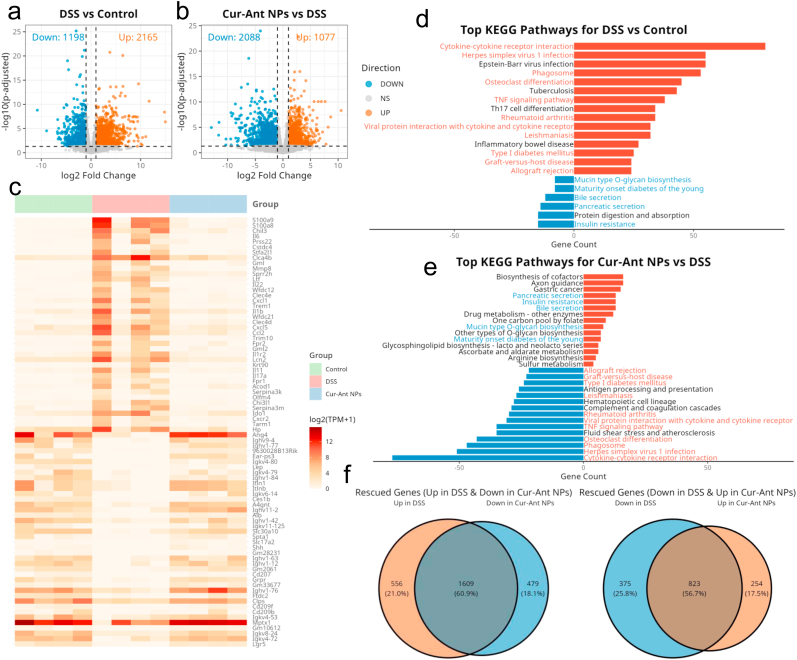
**Transcriptomic Analysis Reveals the Molecular Mechanisms by which Cur-Ant NPs Ameliorate DSS-Induced Colitis.** (a, b) Volcano plots showing the differentially expressed genes (DEGs) in the colon for the comparisons of (a) DSS vs Control and (b) Cur-Ant NPs vs DSS. Red dots represent significantly upregulated genes, and blue dots represent significantly downregulated genes. (c) Heatmap from RNA-seq analysis indicating the response of top differentially expressed genes in the Control, DSS, and Cur-Ant NP-treated groups (log2 (TPM+1) scale on the right). (d, e) Kyoto Encyclopedia of Genes and Genomes (KEGG) pathway enrichment analysis of the DEGs for (d) DSS vs Control and (e) Cur-Ant NPs vs DSS. (f) Venn diagrams illustrating the gene expression reversed by Cur-Ant NP treatment. Left: Genes upregulated in the DSS group but downregulated by Cur-Ant NPs (Rescued: 1609 genes). Right: Genes downregulated in the DSS group but upregulated by Cur-Ant NPs (Rescued: 823 genes). (For interpretation of the references to color in this figure legend, the reader is referred to the Web version of this article.)

To delineate the molecular mechanism of Cur-Ant NPs in ameliorating colitis, we performed a comprehensive transcriptomic analysis of the colon tissues. Principal component analysis (PCA) of the transcriptomic data visualized the global gene expression landscape across the three groups (Fig. S6a). A clear segregation was observed between the DSS and Control groups, confirming a significant disease-induced transcriptomic shift. Notably, the Cur-Ant NPs treatment effectively ameliorated this effect, repositioning the treatment group to cluster tightly with the Control group. This robustly demonstrates the capacity of Cur-Ant NPs to reverse the pathological transcriptomic profile at a global level (PC1 29%, PC2 17%). To identify the specific genes driving these changes, we performed differential expression analysis. Compared to the Control group, DSS treatment induced 3363 differentially expressed genes (DEGs), with 2165 up-regulated and 1198 down-regulated (Fig. 8a). Notably, treatment with Cur-Ant NPs resulted in 3165 DEGs when compared to the DSS group, among which 2088 genes were down-regulated, suggesting a potent suppressive effect on the DSS-induced gene signature (Fig. 8b).

Hierarchical clustering of the top 50 DEGs revealed that DSS induced a distinct pathological gene expression signature, characterized by the strong upregulation of pro-inflammatory genes (e.g., *S100a9*, *Cxcl1*, *Il-1b*) and the concomitant downregulation of genes essential for intestinal homeostasis (e.g., *Ang4*, *Lgr5*) (Fig. 8c). The Cur-Ant NPs treatment almost reversed these transcriptomic alterations. It not only suppressed the DSS-induced inflammatory gene expression but also restored the expression of key homeostatic genes, resulting in a profile that mirrored the healthy Control group. To elucidate the biological pathways and molecular mechanisms underlying the therapeutic efficacy of Cur-Ant NPs, we conducted KEGG enrichment analysis on the DEGs. First, we established the transcriptomic signature of the DSS-induced pathology by comparing the DSS and Control groups (Fig. 8d). This revealed a massive enrichment of pathways associated with inflammation and immunity, including ‘Cytokine-cytokine receptor interaction’, ‘TNF signaling pathway’, and ‘Th17 cell differentiation’ [83]. Concurrently, pathways critical for physiological gut function, such as ‘Protein digestion and absorption’ and ‘Bile secretion’, were significantly suppressed, indicating a severe disruption of metabolic homeostasis. Remarkably, the KEGG analysis for the Cur-Ant NPs vs. DSS comparison demonstrated a comprehensive reversal of this disease phenotype (Fig. 8e). Notably, the enrichment of the ‘Gastric cancer’ pathway (Fig. 8e) reflects the modulation of shared signaling hubs, such as PI3K-Akt and NF-ĸB, which are central to both cancer and IBD. Rather than an oncogenic risk, this enrichment suggests a chemopreventive or protective effect on the mucosa, consistent with the established bioactivities of Cur and Ant [84,85]. The previously upregulated inflammatory and immune-related pathways were profoundly downregulated by our nanoparticle treatment, becoming the most significantly suppressed pathways. In parallel, the metabolic and digestive pathways that were impaired in the disease state were significantly upregulated, indicating a robust restoration of colonic physiological function. Taken together, these results strongly suggest a dual mechanism of action for Cur-Ant NPs: they not only potently suppress the aberrant inflammatory cascade but also actively promote the recovery of metabolic homeostasis. Gene Ontology (GO) analysis further elucidated the dual therapeutic action of our nanoparticles at a functional level (Fig. S6c and d). The treatment profoundly suppressed key Biological Processes (BP) related to inflammation, such as ‘regulation of inflammatory response (GO: 0006954) ' and ‘immune response (GO:0045,087) '. This functional suppression of immunity was corroborated by the downregulation of inflammation-associated Cellular Components (CC) (e.g., ‘collagen-containing extracellular matrix (GO:0062,023) ') and Molecular Functions (MF) (e.g., ‘cytokine receptor binding (GO:0005126) '). Conversely, GO terms related to physiological functions, including epithelial cell components like ‘brush border (GO:0005903) ' (CC) and ‘transmembrane transporter activity (GO:0022,857) ' (MF), were significantly upregulated, indicating a restoration of intestinal homeostasis [18,83].

To provide a quantitative measure of the transcriptomic restoration, we analyzed the overlap between genes dysregulated by DSS and those reciprocally modulated by Cur-Ant NPs. The results demonstrated a profound normalization of the colitic gene signature (Fig. 8f). Specifically, of the 2165 genes upregulated in the DSS model, a remarkable 60.9% (1609 genes) were significantly downregulated by our treatment. Concurrently, 56.7% of the 1198 genes suppressed by DSS were restored to higher expression levels (823 genes). This robust, bidirectional rescue of the majority of disease-associated genes underscores the potent capacity of Cur-Ant NPs to precisely reverse the pathological transcriptome and re-establish molecular homeostasis.

### 5.5 Modulation of Cur-Ant NPs on the gut microbiota

**Fig. 9:**
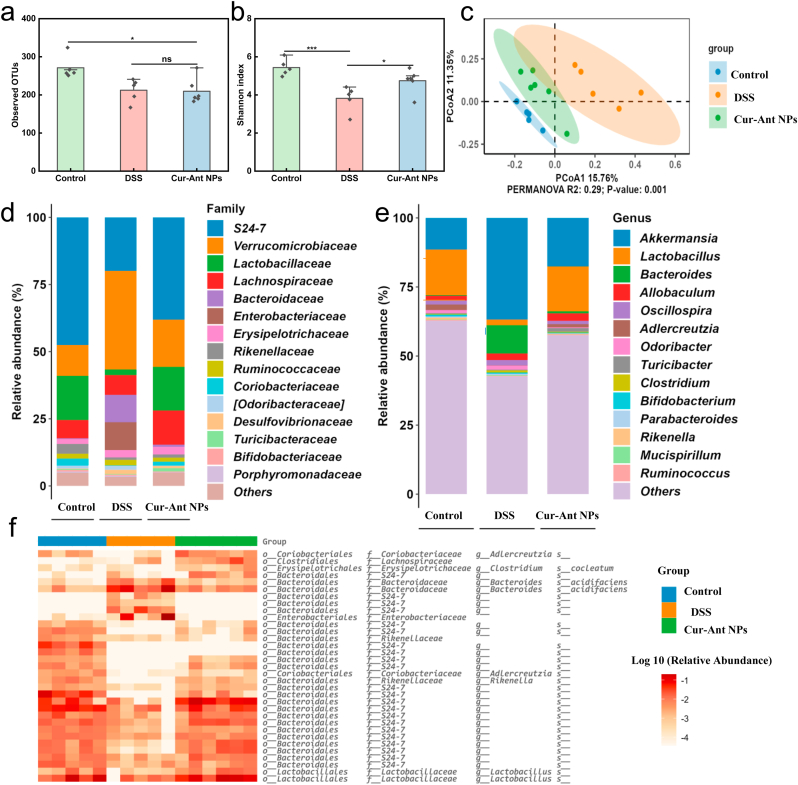
**Cur-Ant NPs Remodel the Gut Microbiota Composition in DSS-Induced Colitis Mice.** (a, b) Alpha diversity analysis of the gut microbiota based on (a) Observed OTUs and (b) the Shannon index. (c) Principal Coordinates Analysis (PCoA) score plot illustrating the beta diversity and structural shifts of the gut microbiota among groups. The variation explained by PCoA1 and PCoA2 is 15.76% and 11.35%, respectively (PERMANOVA *p* = 0.001). (d, e) Stacked bar charts displaying the relative abundance of dominant bacterial taxa at the (d) family and (e) genus levels. (f) Heatmap profiling the differential bacterial taxa (from order to species levels) across individual samples. The color scale represents the Log10-transformed relative abundance. Data in (a, b) are presented as mean ± SD (n = 5–6 mice per group). Statistical significance was determined by one-way ANOVA. ∗*p* < 0.05, ∗∗*p* < 0.01, ∗∗∗*p* < 0.001. Ns indicates no significant difference. (For interpretation of the references to color in this figure legend, the reader is referred to the Web version of this article.)

To investigate the intricate mechanisms by which Cur-Ant NPs confer therapeutic benefits, we performed 16 S rDNA sequencing to characterize the dynamic changes in the gut microbial landscape. Our results reveal that DSS induction precipitates a state of profound gut dysbiosis, which was observed to be notably mitigated following the Cur-Ant NPs intervention, thereby re-establishing microbial homeostasis. First, we assessed the alterations in microbial diversity. DSS treatment led to a significant reduction in alpha diversity, as evidenced by the decreased Observed OTUs, Shannon index, Faith's PD, and Pielou's evenness compared to the healthy control group (Fig. 9a and b, S7a, S7b). Treatment with Cur-Ant NPs, however, was associated with a significant restoration of this loss of diversity, elevating all alpha diversity metrics to levels comparable with, or even exceeding, the control group. Furthermore, PCoA based on beta diversity metrics demonstrated a distinct clustering of the DSS group away from the control group, confirming a significant structural shift in the gut microbial community (PERMANOVA, p < 0.001) (Fig. 9c–S7c). The Cur-Ant NP-treated group, in contrast, clustered closely with the control group, indicating that the nanoformulation facilitated a shift in the microbial composition from a dysbiotic state back towards a healthy profile.

At the taxonomic level (Fig. 9d), DSS induced a dramatic restructuring of the gut microbiota. It triggered a bloom of pro-inflammatory families, notably Enterobacteriaceae, while concurrently depleting crucial beneficial families such as Lactobacillaceae, Rikenellaceae and Muribaculaceae (Former name: S24-7 family). It should be noted that Muribaculaceae, a well-known bacterial family in the human intestinal flora, has been positively associated with metabolic homeostasis through short-chain fatty acid production [86]. Furthermore, this bacterial family ferments dietary fiber to ameliorate intestinal dysbiosis, playing a pivotal role in energy harvest regulation and gut barrier fortification (Fig. S7d) [[87], [88], [89]]. These alterations characterize the disrupted microecosystem of the gastrointestinal tract and are frequently observed in various gastrointestinal-related diseases. The Cur-Ant NP treatment was accompanied by a notable counteraction of this dysbiotic shift. It suppressed the expansion of Enterobacteriaceae and fostered the recovery of beneficial taxa. This was particularly evident at the genus level (Fig. 9e), where the Cur-Ant NPs intervention was linked to the enrichment of key beneficial microbes, including *Lactobacillus* and *Adlercreutzia*. Most notably, we observed an opportunistic bloom of *Akkermansia*, a key mucin-degrading bacterium, in the DSS-induced colitis group compared to healthy controls (Fig. S7e). While often considered beneficial, its role is highly context-dependent. In the pathological setting of DSS-induced colitis where the epithelial barrier is already compromised, this excessive proliferation of a mucin-degrading specialist could be detrimental, potentially exacerbating barrier erosion [90]. The subsequent normalization of *Akkermansia* abundance to control levels after Cur-Ant NPs treatment strongly suggests that our intervention helped restore microbial homeostasis, which closely correlated with the alleviation of colonic inflammation. Moreover, *Lactobacillus*, a well-known probiotic genus in the human intestinal flora, has been positively associated with the enhancement of gut barrier integrity and mucosal immunity. Furthermore, this bacterial genus produces metabolites that ameliorate intestinal dysbiosis, playing a pivotal role in inflammation reduction and epithelial tissue repair (Fig. S7f) [91,92]. A heatmap analysis further confirmed the broad-scale ability of Cur-Ant NPs to reverse the microbial signature associated with DSS induction, elevating beneficial genera like f_Muribaculaceae, g_*Lactobacillus* and *g_Adlercreutzia* while reducing opportunistic pathogens such as f_Enterobacteriaceae, and o_Enterobacterales (Fig. 9f).

In conclusion, these findings collectively suggest that the therapeutic effects observed with Cur-Ant NPs are closely associated with their influence on the colonic microbial milieu. By being accompanied by the restoration of microbial diversity, the suppression of opportunistic pathogens, and the specific enrichment of crucial keystone species, the nanoformulation effectively correlates with the mitigation of gut dysbiosis. This remodeling of the gut microbiota may represent an important pathway contributing to the alleviation of colonic inflammation, highlighting the potential of Cur-Ant NPs as a promising strategy for the management of IBD.

## 6 Conclusions

In summary, we have successfully engineered a carrier-free nanotherapeutic system (Cur-Ant NPs) via the supramolecular self-assembly of curcumin and anthocyanin. Driven by robust π-π stacking and hydrogen bonding interactions, this “green” assembly strategy effectively circumvents the intrinsic solubility and stability limitations of free polyphenols, yielding a biocompatible nanomedicine with superior structural integrity. In vivo evaluations demonstrated that oral administration of Cur-Ant NPs exerts potent therapeutic efficacy against DSS-induced colitis through a synergistic, multi-targeted mechanism: (i) breaking the vicious oxidative-inflammatory cycle by scavenging ROS and suppressing pro-inflammatory cascades; (ii) reinforcing the intestinal epithelial barrier by restoring tight junction proteins (ZO-1 and Occludin); and (iii) remodeling the gut microenvironment by reversing microbial dysbiosis. Despite these promising findings, we acknowledge that this proof-of-concept study primarily focuses on acute therapeutic intervention. Future research is warranted to conduct longitudinal toxicity assessments (e.g., 28-day repeated dose) and evaluate long-term microbial resilience post-treatment to fully elucidate the platform's chronic safety profile and its impact on the persistent stability of commensal bacteria. Collectively, this work not only provides a promising, safe candidate for IBD management but also establishes a versatile, scalable engineering paradigm for the rational design of next-generation supramolecular nanomedicines derived from natural bioactive agents.

## CRediT authorship contribution statement

**Qiwen Xie:** Data curation, Investigation, Writing – original draft, Writing – review & editing. **Huan Xu:** Conceptualization, Data curation, Resources, Software, Validation. **Xiaoming Yang:** Data curation, Resources, Software, Supervision. **Ying Chen:** Investigation, Methodology, Project administration, Resources, Software. **Zhenjiang Zech Xu:** Funding acquisition, Project administration, Resources, Writing – original draft, Writing – review & editing.

## Declaration of competing interest

The authors declare that they have no known competing financial interests or personal relationships that could have appeared to influence the work reported in this paper.

## Data Availability

Data will be made available on request.
