# Supplementary material for: Natural carrier-free self-assembled binary polyphenol nanoparticles remodel the gut microenvironment for inflammatory bowel disease prevention
Source: Mater Today Bio. 2026 Mar 28;38:103063. doi: 10.1016/j.mtbio.2026.103063 (PMC13087755; doi:10.1016/j.mtbio.2026.103063)

**Supplementary information**

**Natural Carrier-Free Self-Assembled Binary Polyphenol Nanoparticles Remodel the Gut Microenvironment for Inflammatory Bowel Disease Prevention**

Qiwen Xie **^†^**, Huan Xu**^†^**, Xiaoming Yang**^†^**, Ying Chen**^†^**, Zhenjiang Zech Xu**^†,‡,*^**

**^†^**State Key Laboratory of Food Science and Resources, Nanchang University, Nanchang 330047, China.

**^‡^** Lead contact.

**^*^**Correspondence: zhenjiang.xu@gmail.com.

**Supporting experimental procedures**

**Evaluation of Aqueous Dispersibility**

Excess Cur-Ant NPs and Free Cur were dispersed in 5 mL of PBS (pH 2.0 and 7.4), deionized water, and 1% (v/v) DMSO via sonication. Aliquots (20 μL) were withdrawn immediately (t = 0 h) and after 2 h of undisturbed sedimentation (from the supernatant), then fully dissolved in 180 μL methanol. Absorbance was measured at 427 nm. The dispersibility retention was calculated as (A_3h_/A_0h_) × 100%.

**Thermal Stability, UV Exposure, and pH Stability Studies**

Cur-Ant NPs were dissolved in PBS buffer at pH 7.4 and heated in a water bath at 50, 60, 70, 80, and 90°C for 60 minutes. For the evaluation of UV exposure stability, sample solutions in transparent glass vials were stored in a darkroom and exposed to a 30 W UV lamp for 4, 8, 12, 16, and 24 hours. The pH stability study was conducted by dispersing Cur-Ant NPs and Free Cur in PBS (10 mM, pH 8) for 0, 1, 4, 8, and 12 hours, followed by the determination of residual drug content via UV-Vis absorption spectroscopy at 430 nm [1].

**Molecular dynamics simulation**

The molecular dynamics simulations were performed using the GROMACS 2020.6 package [2]. The Visualization of structure was performed by VMD software. Two separate systems were prepared: one with 10 Cur and 10 Ant molecules, and another with 5 Cur and 15 Ant molecules. These molecules were randomly mixed in a 5 × 5 × 5 nm cubic box with periodic boundary conditions by using PACKMOL package. The ACPYPE code was used to generate the desired force field parameters for the simulation systems. The partial charges on atoms were obtained using restrained electrostatic potential (RESP) method which calculated with Multiwfn software. Before starting MD simulation, the initial configurations were relaxed using a conjugate gradient minimization scheme. The step size was 0.01 nm, and the cycle was set to 5000 steps. Minimization was considered converged when the minimum force was less than 50 kJ·mol^-1^·nm^-1^. The van der Waals interaction was calculated by the cut-off method, atomic electrostatic interaction was calculated by PME (particle mesh Ewald), and both the cut-off and PME distances were 1.0 nm. Then, the system was equilibrated with a pressure of 1.0 bar to achieve a desired density. The Berendsen and V-rescale methods were used to control the pressure and temperature. The time constant was 1.0 ps, and the compressibility was 4.5 × 10^−5^ bar^−1^. The equilibrium was 5 ns for all systems with a 0.001 ps time step. Finally, the production ran for 100 ns. The pressure control was changed to the Parrinello-Rahman method in the production run. In addition, the LINCS (Linear Constrain Solver) algorithm was used to impose constraints on the hydrogen bond. The data were analysed through the Gromacs embedded section [1, 3].

**In Vitro Cur release**

The in vitro release profile of Cur from Cur-Ant NPs was determined by ultraviolet-visible (UV-Vis) absorption spectroscopy, leveraging the intrinsic absorption characteristics of Cur. Specifically, dialysis bags (MWCO: 3500, Spectrum Laboratories) containing 2 mL of Cur-Ant NP suspension (1 mg/mL) were immersed in 20 mL PBS buffers (pH 7.4 simulating colonic microenvironment or pH 2 mimicking gastric microenvironment, supplemented with 0.5% Tween 20) and incubated at 37 ℃ for 72 h (n = 3). Aliquots (1 mL) of supernatant were collected at predetermined time intervals (0.5, 1, 2, 4, 6, 8, 12, 24, 36, 48, and 72 h), with immediate replenishment of 1 mL fresh PBS at corresponding pH values. To calculate cumulative drug release ratios, free Cur treated with PBS (pH 2 containing 0.5% Tween 20) for 72 h was defined as 100% release.

**Zebrafish husbandry**

Zebrafish (*Danio rerio*) *Tg (lyz:DsRed2*) transgenic lines (obtained from the National Zebrafish Resource Center) and wild type (AB) were used in this study. All fish were reared and maintained at 28 ± 1 ℃ with a 14 h light: 10 h dark cycle (light on at ZT0 and light off at ZT14). Adult zebrafish were placed in the mating tank on the previous day and separated by a partition board (the ratio of male to female was 1:1 or 1:2). After entering the photoperiod on the second day, the isolation board was removed and embryos were collected [4]. To avoid the formation of pigments, the larvae were kept in embryo medium supplemented with 0.2 mM 1-phenyl-2-thiourea. Breeding was carried out following standard experimental protocols in compliance with the guidelines of the laboratory animal management committee.

**Zebrafish embryo toxicity test**

The method was adopted from previous toxicity assays with slight modifications [4-6]. Acute toxicity test of the zebrafish larva: The free Cur, Ant and Cur-Ant NPs were dissolved in the embryonic medium containing 0.1% DMSO to prepare well-mixed solutions containing 50, 100, 300, 500, and 1000 μg/L concentrations. The 3 dpf zebrafish were co-incubated with NPs of different concentrations in 6-well plates, and 30 zebrafish larvae in each well were immersed in 5 mL solution as a parallel, with three parallels in each group. Fresh liquid and NPs were changed daily, and the survival rate was recorded for 96 h. Embryo incubation experiment: Solutions were prepared (50, 100, 300, 500 and 1000 μg/L) by dissolving NPs in the embryonic medium and stirring well. The NPs solution was co-incubated with 2 hpf zebrafish embryos in 6-well plates, and 30 zebrafish embryos in each well were immersed in 5 mL solution as a parallel, with three parallels in each group. Fresh liquid and BGs were changed daily, and the hatching rate was recorded at 3 dpf. All stock solutions ensured that the concentration of DMSO in the experimental exposure solution did not exceed 0.1%, a concentration reported to be non-toxic to zebrafish.

**Establish a zebrafish model of IBD**

At 3 days post-fertilization (dpf), AB strain zebrafish or transgenic *Tg* (*lyz:DsRed*2) larvae were randomly divided into six groups (n = 30 per group): a control group, a 0.4% DSS model group, and four treatment groups receiving 0.4% DSS co-administered with either SASP, Free Ant, Free Cur, or Cur-Ant NPs. The concentration for all drug treatments was maintained at 500 μg/L.

Subsequently, the larvae were transferred to 6-well plates and immersed in embryonic medium containing the respective formulations. The therapeutic agents were internalized primarily through swallowing and gill absorption. The medium containing fresh drugs was renewed daily, and relevant indicators were assessed at 8 dpf.

**ROS imaging**

According to our previous report [4, 6, 7], The ROS in live zebrafish cells were detected using the fluorescent probe 2′,7′-dichloro-fluorescein diacetate (DCFH-DA, Nanjing Jiancheng Bioengineering Institute, E004-1-1). Zebrafish larval samples were collected, DCFH-DA (excitation wavelength: 488 nm; emission wavelength: 525 nm) was added until the final concentration was 10 μM, and then it was left to incubate for 30 minutes in the dark at a constant temperature of 28 °C. After that, the zebrafish larvae were washed with fish solution three times and immersed in 0.016% Tricaine anesthetic. A fluorescence microscope (ZEISS, AXIO Zoom. V16) was used to observe and record the images. We used ZEISS software to measure the fluorescence intensity of the ROS staining data. 10 zebrafish larvae in each group, with three independent experiments. Finally, the total fluorescence intensity (TFI) of green light emitted from the intestines of zebrafish was calculated using Image J (National Institutes of Health, USA).

**Migration of intestinal immune cells**

Zebrafish embryos of *Tg(lyz:DsRed2*) transgenic lines were collected according to the required mating method. The EGFP marked macrophages and the *DsRed* marked neutrophils in *Tg(lyz:DsRed2*) transgenic lines. After administration as previously described, anesthetized all groups of 8 dpf larval zebrafish (zebrafish were immersed in 0.016% Tricaine anesthetic), fixed with 1% low melting point agarose, and photographed by ZEISS stereo fluorescence microscope (AXIO Zoom. V16) as soon as possible. 15 zebrafish larvae in each group, with three independent experiments. The number of red neutrophils in the intestines of zebrafish was calculated using Image J.

**Quantitative real-time polymerase chain reaction**

A total of 100 zebrafish larvae were placed in a 2 mL centrifuge tube as a biological replicate, with three replicates per group. The total RNA of the fish larva was extracted using TRIzol one-step extraction (Trizol reagent; Invitrogen, USA) and reverse transcribed into cDNA using HiScript® II Q RT SuperMix for qPCR kit. The qPCR was performed using the SYBR PCR master kit (Thermo Fisher, USA). Primers at 5′-3′ are showed in **Table S1**. The comparative threshold cycle CT method (2^-△△CT^) with β-actin as the internal reference gene was used to calculate RNA relative expression as fold alteration. The expression level of the control group is 1, and the relative mRNA expression level in the treated samples is its multiple [6].

**Oxidative stress analysis**

The Nanjing Jiancheng Bioengineering Institute (Nanjing, China) provided test kits for measuring the enzyme activity or other biological indicators. To determine how much oxidative stress zebrafish larvae were under, the manufacturer's method was used to measure the levels of malondialdehyde (MDA, A003-1-2), catalase (CAT, A007-1-1), and superoxide dismutase (SOD, A001-3-2). The absorbance was quantified using a SpectraMax iD3 multi-mode microplate reader (USA) and normalized to the total protein levels. 30 zebrafish larvae in each group, with three independent experiments [4, 7].

**Hematoxylin and eosin (H&E) staining**

Several zebrafish larvae following a 5-day period of drugs were collected from the different groups and washed thrice with PBS for 5 minutes and kept in overnight incubation with 4% paraformaldehyde solution (PFA) at 4 °C. After dehydration with an ethanol gradient, the fixed larvae were embedded in paraffin wax and sliced into 5 μm sections using a Leica microtome. The H&E staining procedures were carried out according to the previously protocols, and images were observed and photographed with a microscope (Leica DM2500, Germany). 5 zebrafish larvae in each group, with three independent experiments [6, 7].

**16S rRNA gene sequencing analysis**

For microbiota analysis, whole 100 zebrafish larvae were collected, flash-frozen in liquid nitrogen, and shipped on dry ice to Novogene Co., Ltd. (Beijing, China) for all subsequent processing. The sequencing library was constructed, paired-end sequences were merged, and the data were qualityfiltered, ensuring the reliability of subsequent analyses. Finally, sequences with a similarity of 97% were clustered into operational taxonomic units (OTU). Alpha (α)-diversity and OTU abundance curve analyses were performed at the OTU level. The composition of the intestinal flora was analyzed at the phylum, genus, and species levels. Detailed procedures can follow 16S rRNA sequencing of mice fecal.

**Animal study on mice**

Male C57BL/6 mice ((Male, 7-8 weeks, 22 ± 2 g) were obtained from GemPharmatech Co., Ltd. (Nanjing, China). The rats were fed adaptively for 7 d in a standardized animal room (temperature 25±1 ℃, relative humidity 40–70%, dark/light cycles 12 h, sufficient water supply and feed). All animal experiments were conducted in accordance with the National Regulation of China for Care and Use of Laboratory Animals. The experimental protocols involving live animals were reviewed and approved by the Animal Ethics Committee of the Nanchang University (ethical code: SYXK (Gan) 2021–0004).

**DSS Induced IBD mice model establishment**

To evaluate the biological activity of Cur-Ant NPs in vivo, mice received dextran sodium sulfate (DSS, 40 kDa, MP Biomedical) in their drinking water to induce IBD during 7 consecutive days. A total of 48 male C57BL/6 mice were randomly allocated into six groups (n=8 per group): Control, DSS, DSS + SASP, DSS + Free Ant, DSS + Free Cur, and DSS + Cur-Ant NPs groups. Throughout the experiment, the mice in the control group consumed diet normally. The model group of mice consumed normal diet for the first 3 days before receiving 2.5% DSS (w/v) [8]. The other groups received 2.5% DSS on day 3, a daily dose of 10 mg kg^-1^ of different drugs for the following 10 days. Throughout the experiment, the Disease Activity Index (DAI) was monitored daily to assess disease progression. The DAI score, which combines weight loss, stool consistency, and rectal bleeding, was calculated based on the criteria outlined in **Table S2** [3, 9].

**ELISA test of cytokines**

The concentrations of interleukin-6 (IL-6), interleukin-10 (IL-10), and tumor necrosis factor-alpha (TNF-α) in colon tissue homogenates were quantified using commercial enzyme-linked immunosorbent assay (ELISA) kits (Servicebio Biotech, Wuhan, China). All assays were performed strictly according to the manufacturer’s protocols. The optical density at 450 nm was measured using a Tecan Infinite M200 Pro microplate reader. Cytokine concentrations were calculated based on the standard curves generated for each assay. A detailed step-by-step procedure is provided in the Supporting Information.

**Western blot**

To determine the protein expression levels of key tight junction proteins, Western blot analysis was performed. Total protein was extracted from frozen colon tissues by homogenization in ice-cold RIPA lysis buffer (Servicebio Biotech, Wuhan, China) supplemented with a 1% protease inhibitor cocktail (e.g., PMSF, Servicebio Biotech). The resulting protein lysates were cleared by centrifugation at 12,000 × g for 15 minutes at 4 °C. Protein concentration was quantified using a bicinchoninic acid (BCA) protein assay kit (Servicebio Biotech) according to the manufacturer's protocol. For each sample, 40 µg of total protein was resolved by 10% SDS-polyacrylamide gel electrophoresis (SDS-PAGE) and subsequently transferred onto polyvinylidene fluoride (PVDF) membranes (Millipore, Billerica, MA, USA). The membranes were blocked for 2 hours at room temperature in 5% (w/v) non-fat dry milk prepared in Tris-buffered saline with 0.1% Tween 20 (TBST). Following blocking, membranes were incubated overnight at 4 °C with the following primary antibodies: rabbit anti-ZO-1, rabbit anti-OCCLUDIN and Mouse anti-β-ACTIN. After three washes with TBST, the membranes were incubated with the appropriate horseradish peroxidase (HRP)-conjugated secondary antibodies for 1 hour at room temperature. Protein bands were visualized using an enhanced chemiluminescence (ECL) detection kit (Servicebio Biotech) and imaged on a. Densitometric analysis of the bands was performed using Image Lab software (Bio-Rad), with β-ACTIN serving as the internal loading control.

**Histological and histochemical analysis**

To evaluate colon morphology and goblet cell populations, standard histological and histochemical staining was performed. Immediately following euthanasia, murine colons were excised, flushed with cold PBS to remove contents, and fixed in 4% paraformaldehyde for 24 hours at 4 °C. For paraffin embedding, the fixed tissues underwent a standard processing protocol: dehydration through a graded series of ethanol (75%, 85%, 95%, and 100%), followed by clearing in two changes of xylene. The processed tissues were then embedded in paraffin blocks and sectioned to a thickness of 4 µm. For analysis, sections were deparaffinized and rehydrated. General tissue morphology was assessed using a Hematoxylin and Eosin (H&E) staining kit (Solarbio, G1100). Goblet cells and mucin production were visualized with an Alcian Blue-Periodic Acid Schiff (AB-PAS) staining kit (Solarbio, G1285), following the manufacturer’s instructions. High-resolution digital images of the stained sections were acquired using a NanoZoomer S60 digital pathology scanner (Hamamatsu). Histological scoring was conducted based on the criteria detailed in **Table S3** [3].

**ROS Measurement**

ROS Measurement Freshly harvested colon tissues were immediately embedded in optimal cutting temperature (OCT) compound and snap-frozen in liquid nitrogen. To preserve the reactive oxygen species (ROS) levels, the samples were sectioned into 20 μm cryosections using a Leica cryostat at -20 ℃. For ROS detection, the sections were incubated with 5 μM dihydroethidium (DHE; Beyotime Biotechnology, China) in a dark, humidified chamber for 30 min at 37 ℃, according to the manufacturer's protocol. The nuclei were counterstained with 4',6-diamidino-2-phenylindole (DAPI; Beyotime Biotechnology). Fluorescence images were acquired using an Olympus confocal laser scanning microscope.

**Immunofluorescence**

The spatial expression of OCCLUDIN and ZO-1 in murine colon tissue was evaluated by immunofluorescent staining. Paraffin-embedded colon sections (4 µm thick) were deparaffinized in xylene and rehydrated through a graded ethanol series. Antigen retrieval was performed by heating the slides in at 95 °C for 15 minutes. To block nonspecific antibody binding, the sections were permeabilized with for 10 minutes and subsequently incubated with 5% normal goat serum for 1 hour at room temperature. The sections were then incubated overnight at 4 °C with the following primary antibodies: rabbit anti-OCCLUDIN and rabbit anti-ZO-1. After washing three times in PBS, the sections were incubated with an Alexa Fluor 488-conjugated goat anti-rabbit IgG secondary antibody for 1 hour in the dark at room temperature. Nuclei were counterstained with 4′,6-diamidino-2-phenylindole (DAPI). Finally, the slides were mounted with an anti-fade mounting medium. Images were captured and analyzed using a fluorescence microscope.

**Immunohistochemistry**

Immunohistochemistry was performed to evaluate the expression and localization of myeloperoxidase (MPO) in colon tissues1. Briefly, paraffin-embedded colon sections (4 μm thickness) were deparaffinized in xylene and rehydrated through a graded series of ethanol. Antigen retrieval was conducted using a citrate antigen retrieval buffer (pH 6.0) in a microwave oven (8 min at medium heat, followed by 7 min at medium-low heat). To quench endogenous peroxidase activity, sections were incubated with 3% H_2_O_2_ for 25 min at room temperature in the dark. After washing with PBS, nonspecific binding was blocked with 3% BSA for 30 min. The sections were then incubated with the primary antibody against MPO overnight at 4 °C in a humidified chamber. Following three washes with PBS, the sections were incubated with an HRP-labeled secondary antibody for 50 min at room temperature. Immunoreactivity was visualized using a DAB chromogen kit, where MPO-positive expression was identified by a brownish-yellow color. Subsequently, sections were counterstained with hematoxylin for 3 min to label cell nuclei. Finally, the slides were dehydrated through graded alcohols, cleared in xylene, and mounted with neutral resin. Images were captured and analyzed using an optical microscope (Eclipse Ci-e, Nikon, Japan).

**RNA Extraction, library construction, and sequencing**

Total RNA was isolated from murine colon tissues using TRIzol® Reagent (Invitrogen, Carlsbad, CA, USA) and treated with DNase I (TaKara, Dalian, China) to remove genomic DNA contamination. The integrity and purity of the extracted RNA were assessed using an Agilent 2100 Bioanalyzer with the RNA Nano 6000 Assay Kit (Agilent Technologies, Santa Clara, CA, USA). Only samples with an RNA Integrity Number (RIN) ≥ 8.0, an OD260/280 ratio of 1.8–2.2, and an OD260/230 ratio ≥ 2.0 were used for subsequent library construction. RNA concentration was quantified using a NanoDrop spectrophotometer (Thermo Fisher Scientific, Waltham, MA, USA).

Sequencing libraries were prepared from 1 μg of total RNA per sample by Novogene Co., Ltd. (Beijing, China) using the TruSeq Stranded mRNA Library Prep Kit (Illumina, San Diego, CA, USA). Briefly, poly (A) mRNA was enriched using oligo (dT) magnetic beads and then fragmented. First-strand cDNA was synthesized using random hexamer primers, followed by second-strand synthesis. The resulting double-stranded cDNA fragments were end-repaired, A-tailed, and ligated to Illumina indexing adapters. Ligated fragments of approximately 300 bp were size-selected on a 2% agarose gel. The final libraries were amplified via a 15-cycle PCR using Phusion High-Fidelity DNA Polymerase (New England Biolabs, Ipswich, MA, USA) and their quality was confirmed with an Agilent 2100 Bioanalyzer. The libraries were then sequenced on an Illumina NovaSeq 6000 platform to generate 150 bp paired-end reads, yielding a minimum of 40 million raw reads per sample.

**Bioinformatic analysis**

Raw sequencing reads were first processed to ensure high quality. Adapters were trimmed using SeqPrep (v1.1), and low-quality bases (Phred score < 20) were removed using Sickle (v1.33). The resulting clean reads were aligned to the Mus musculus reference genome (GRCm39) using HISAT2 (v2.2.1) in a strand-specific mode. Gene expression levels were quantified as Transcripts Per Million (TPM) using RSEM (v1.3.3). Differential expression analysis between experimental groups was performed with the R package DESeq2 (v1.34.0). Genes with an absolute log₂ (fold change) > 1 and a false discovery rate (FDR) adjusted p-value < 0.05 were considered differentially expressed genes (DEGs). To investigate the biological functions of these DEGs, Gene Ontology (GO) and Kyoto Encyclopedia of Genes and Genomes (KEGG) pathway enrichment analyses were conducted using Goatools (v1.2.3) and KOBAS (v3.0), respectively. A Bonferroni-corrected p-value < 0.05 was set as the threshold for significantly enriched terms [3].

**16S rRNA sequencing of mice fecal and analysis**

Fresh fecal samples were collected from each mouse into sterile cryovials, immediately flash-frozen in liquid nitrogen, and stored at -80 °C until shipment on dry ice. All subsequent processing, including DNA extraction and sequencing, was performed by Novogene Co., Ltd. (Beijing, China). Total microbial genomic DNA was extracted from 200 mg of each fecal sample using the E.Z.N.A.® Soil DNA Kit (Omega Bio-tek, Norcross, GA, USA) according to the manufacturer's protocol. The concentration and purity of the extracted DNA were determined using a NanoDrop 2000 spectrophotometer (Thermo Fisher Scientific), with samples meeting the quality criteria of an A260/A280 ratio between 1.8 and 2.0 and an A260/A230 ratio ≥ 1.7. DNA integrity was further confirmed by 1% agarose gel electrophoresis to verify the presence of high-molecular-weight DNA. The V3-V4 hypervariable regions of the bacterial 16S rRNA gene were amplified using the primer pair 338F (5′-ACTCCTACGGGAGGCAGCAG-3′) and 806R (5′-GGACTACHVGGGTWTCTAAT-3′). PCR amplifications were performed in triplicate for each sample in a 20 μL reaction volume containing 10 ng of template DNA, 4 μL of 5× FastPfu Buffer, 2 μL of 2.5 mM dNTPs, 0.8 μL of each primer (5 μM), and 0.4 μL of FastPfu High-Fidelity Polymerase. The thermal cycling conditions were as follows: an initial denaturation at 95 °C for 3 min, followed by 27 cycles of 95 °C for 30 s, 55 °C for 30 s, and 72 °C for 45 s, with a final extension at 72 °C for 10 min. The triplicate PCR products for each sample were pooled and purified using the AxyPrep DNA Gel Extraction Kit (Axygen Biosciences, Union City, CA, USA). The concentration of the purified amplicons was measured using a QuantiFluor™-ST fluorometer (Promega, Madison, WI, USA). Sequencing libraries were prepared by ligating the amplicons with Illumina-compatible barcoded adapters, and the final library quality was validated. The libraries were pooled in equimolar ratios and sequenced on an Illumina NovaSeq 6000 platform, generating 2 × 250 bp paired-end reads, with a targeted depth of at least 100,000 raw reads per sample. Raw sequencing data were demultiplexed based on sample-specific barcodes and delivered as FASTQ files for downstream bioinformatic analysis.

Bioinformatic analysis of the sequencing data was performed using QIIME 2 [10]. Raw paired-end reads were processed using the DADA2 plugin for quality filtering, denoising, merging, and chimera removal to generate a feature table of amplicon sequence variants (ASVs) [11]. Taxonomic classification was assigned to the ASVs using a pre-trained Naïve Bayes classifier against the SILVA database (v138) clustered at 99% similarity [12]. Alpha diversity metrics (Chao1 and Shannon indices) and beta diversity metrics (Bray-Curtis dissimilarity and weighted/unweighted UniFrac distances) were calculated to assess microbial community structure. Principal Coordinate Analysis (PCoA) was used to visualize differences in microbial community composition, and statistical significance was determined using Permutational Multivariate Analysis of Variance (PERMANOVA). To identify differentially abundant bacterial taxa between experimental groups, the Linear discriminant analysis (LDA) effect size (LEfSe) method was employed, identifying taxa with an LDA score > 2.0 [13]. Finally, Spearman's rank correlation analysis was performed to evaluate the relationships between the relative abundances of key differential bacterial taxa and host inflammatory markers.

**Table supplementary information**

**Table S1.** RT-PCR primer sequence

| Primer | Sequence (5′->3′) | Reference |
| --- | --- | --- |
| *Tnf-α* | F: TCTGAACTGACTGAGGAACAAG  R: AAGTGCTGTGGTCGTGTCT | [14] |
| *Il10* | F: GGAGACCATTCTGCCAACAG  R: CACCATATCCCGCTTGAGTTC | [15] |
| *Il6* | F: GTCTGCTACACTGGCTACACT  R: CACATCCTGAACTTCGTCTCC | [14] |
| *β-actin* | F: TGGCATCACACCTTCTACAATG  R: CACCAGAGTCCATCACAATACC | [14] |

**Table S2. Disease activity index（DAI）scoring**

| Score | Body weight loss (a) | Stool traits (b) | Stool occult blood (c) |
| --- | --- | --- | --- |
| 0 | None | Normal | - |
| 1 | 1%-5% | Loose stools | + |
| 2 | 6%-10% |  | ++ |
| 3 | 11%-15% |  | +++ |
| 4 | >15% | Watery diarrhea | ++++ |
| DAI= (a + b + c) /3 | | | |

**Table S3. H&E scoring**

| Score | Epithelial Injury (a) | Loss of crypt architecture (b) | Inflammatory Infiltration (c) |
| --- | --- | --- | --- |
| 0 | None | Normal | Absent |
| 1 | Mild | Mild reduction | Focal |
| 2 | Moderate | Marked reduction | Moderate |
| 3 | Severe | Near-complete loss | Extensive |

**References**

1. Hu Y, Miao Y, Zhang Y, Wang X, Liu X, Zhang W, Deng D: **Co-Assembled Binary Polyphenol Natural Products for the Prevention and Treatment of Radiation-Induced Skin Injury.** *ACS Nano* 2024, **18:**27557-27569.

2. Wang Z, Lu J, Yuan Z, Pi W, Huang X, Lin X, Zhang Y, Lei H, Wang P: **Natural Carrier‐Free Binary Small Molecule Self‐Assembled Hydrogel Synergize Antibacterial Effects and Promote Wound Healing by Inhibiting Virulence Factors and Alleviating the Inflammatory Response.** *Small* 2022, **19(5):e2205528.**

3. Gao S, Zheng H, Xu S, Kong J, Gao F, Wang Z, Li Y, Dai Z, Jiang X, Ding X, Lei H: Novel Natural Carrier‐Free Self‐Assembled Nanoparticles for Treatment of Ulcerative Colitis by Balancing Immune Microenvironment and Intestinal Barrier. Advanced Healthcare Materials 2023, 12(31):e2301826.

4. Tang Y, Zhan Y, Gao S, Li T, Xuan H: **Hepatotoxicity of imidacloprid in zebrafish and the alleviating role of 10-hydroxy-2-decenoi acid: Insights into oxidative stress, inflammation, and gut microbiota.** *J Hazard Mater* 2025, **494:**138695.

5. Eissa MA, Hashim YZHY, Mohd Nasir MH, Nor YA, Salleh HM, Isa MLM, Abd-Azziz SSS, Abd Warif NM, Ramadan E, Badawi NM: **Fabrication and characterization of Agarwood extract-loaded nanocapsules and evaluation of their toxicity and anti-inflammatory activity on RAW 264.7 cells and in zebrafish embryos.** *Drug Delivery* 2021, **28:**2618-2633.

6. Chen H, Lei P, Ji H, Ma J, Fang Y, Yu H, Du J, Qu L, Yang Q, Luo L, et al: **Escherichia coli Nissle 1917 ghosts alleviate inflammatory bowel disease in zebrafish.** *Life Sciences* 2023, **329:121956**.

7. Wu Z, Liu L, Li L, Cao X, Jia W, Liao X, Zhao Z, Qi H, Fan G, Lu H, et al: **Oral nano-antioxidants** **improve sleep by restoring intestinal barrier integrity and preventing systemic inflammation.** *National Science Review* 2023, **10(12):nwad309.**

8. Long J, Liu X-K, Kang Z-P, Wang M-X, Zhao H-M, Huang J-Q, Xiao Q-P, Liu D-Y, Zhong Y-B: **Ginsenoside Rg1 ameliorated experimental colitis by regulating the balance of M1/M2 macrophage polarization and the homeostasis of intestinal flora.** *European Journal of Pharmacology* 2022, **917:174742.**

9. Xie Q, Li H, Ma R, Ren M, Li Y, Li J, Chen H, Chen Z, Gong D, Wang J: **Effect of Coptis chinensis franch and Magnolia officinalis on intestinal flora and intestinal barrier in a TNBS-induced ulcerative colitis rats model.** *Phytomedicine* 2022, **97:153927.**

10. Bolyen E, Rideout JR, Dillon MR, Bokulich NA, Abnet CC, Al-Ghalith GA, Alexander H, Alm EJ, Arumugam M, Asnicar F, et al: **Reproducible, interactive, scalable and extensible microbiome data science using QIIME 2.** *Nat Biotechnol* 2019, **37:**852-857.

11. Callahan BJ, McMurdie PJ, Rosen MJ, Han AW, Johnson AJ, Holmes SP: **DADA2: High-resolution sample inference from Illumina amplicon data.** *Nat Methods* 2016, **13:**581-583.

12. Quast C, Pruesse E, Yilmaz P, Gerken J, Schweer T, Yarza P, Peplies J, Glöckner FO: **The SILVA ribosomal RNA gene database project: improved data processing and web-based tools.** *Nucleic Acids Res* 2013, **41:**D590-596.

13. Segata N, Izard J, Waldron L, Gevers D, Miropolsky L, Garrett WS, Huttenhower C: **Metagenomic biomarker discovery and explanation.** *Genome Biol* 2011, **12:**R60.

14. Nayak SPRR, Dhivya LS, R R, Almutairi BO, Arokiyaraj S, Kathiravan MK, Arockiaraj J: **Furan based synthetic chalcone derivative functions against gut inflammation and oxidative stress demonstrated in in-vivo zebrafish model.** *European Journal of Pharmacology* 2023, **957:175994**.

15. Brugman S, Liu KY, Lindenbergh-Kortleve D, Samsom JN, Furuta GT, Renshaw SA, Willemsen R, Nieuwenhuis EE: **Oxazolone-induced enterocolitis in zebrafish depends on the composition of the intestinal microbiota.** *Gastroenterology* 2009, **137:**1757-1767 e1751.

**Figure S1. Supplementary Physicochemical Characterization of Cur-Ant Nanoparticles.** (a) Scanning electron microscopy (SEM) images of the raw starting materials: free Anthocyanin (Ant) and free Curcumin (Cur). (b) In vitro cumulative release profiles of Cur from Free Cur and Cur-Ant NPs in PBS at pH 2.0 (simulating gastric fluid) and pH 7.4 (simulating intestinal fluid). (c) Storage stability of Cur-Ant NPs in room temperature over 12 days, monitored by changes in particle size and polydispersity index (PDI). (d-f) Chemical stability of Cur within Cur-Ant NPs compared to Free Cur under different stress conditions: (d) thermal stress at various temperatures, (e) UV irradiation over 24 hours, and (f) alkaline conditions (pH 9.0) over 12 hours. (g) High-Resolution Mass Spectrometry (HRMS) spectrum of Cur-Ant NPs, showing the characteristic peaks for Ant, Cur, and the non-covalent [Cur+Ant+H]⁺ complex. Data in (b-f) are presented as mean ± SD from three independent experiments.

**Figure S2. Molecular Dynamics Simulations and Antioxidant Activity Optimization.** (a, b) Analysis of the Molecular Dynamics (MD) simulation for the Cur-Ant complex (1:1 molar ratio) over a 25 ns trajectory: (a) Solvent-Accessible Surface Area (SASA) and (b) number of intermolecular hydrogen bonds. (c, d) Corresponding MD simulation analysis for the Cur-Ant complex (1:3 molar ratio) extended to 50 ns: (c) SASA and (d) number of intermolecular hydrogen bonds. (e, f) Screening of the optimal molar ratio based on antioxidant capacity. Comparison of (e) DPPH and (f) ABTS radical scavenging rates among Free Cur, Free Ant, and Cur-Ant NPs prepared at different molar ratios (1:1 to 1:4). Data in (e, f) are presented as mean ± SD (n = 3).

**Figure S3. Biosafety Assessment of Cur-Ant NPs in the Zebrafish Model.** (a, b) In vivo toxicity assessment of the different formulations in healthy zebrafish embryos/larvae. (a) Hatching rate of zebrafish embryos and (b) survival rate of zebrafish larvae after exposure to various concentrations (50, 100, 300, 500, and 1000 μg/L) of Free Ant, Free Cur, and Cur-Ant NPs. Data are presented as mean ± SD. No significant differences were observed compared to the Control group across all tested concentrations.

**Figure S4.** Cur-Ant NPs Modulate the Gut Microbiota in the Zebrafish Model of DSS-Induced Enterocolitis, Related to Figures 3 and 4. (a, b) Alpha diversity analysis of the zebrafish gut microbiota, as measured by (a) Observed OTUs and (b) the Shannon index. (c) Principal Coordinates Analysis (PCoA) plot based on Bray-Curtis dissimilarity, illustrating the structural shifts in the gut microbial communities among the three groups (Control, DSS, and Cur-Ant NPs). (d, e) Relative abundance of the dominant bacterial taxa at the (d) phylum and (e) genus levels. Data in (a, b) are presented as mean ± SD. Statistical significance was determined by one-way ANOVA. **p* < 0.05, ***p* < 0.01. NS indicates no significant difference.

**Figure S5. Quantitative Analysis of Intestinal Barrier Protein Expression in DSS-Induced Colitis Mice, Related to Figure 7.** (a, b) Densitometric quantification of the Western blot bands for (a) ZO-1 and (b) OCCLUDIN. Protein expression levels were normalized to Actin. These quantitative data correspond to the representative Western blot images shown in Figure 7a. Data are presented as mean ± SD ($n = 3$ independent experiments). Statistical significance was determined by one-way ANOVA. ****p* < 0.001.

**Figure S6. Functional Enrichment Analysis of Differentially Expressed Genes in Colon Tissues, Related to Figure 8.** (a) Principal Component Analysis (PCA) plot of the transcriptomes from colon tissues of the Control, DSS, and Cur-Ant NP-treated groups ($n = 4$ per group), showing distinct clustering based on gene expression profiles. (b, c) Gene Ontology (GO) enrichment analysis of the differentially expressed genes (DEGs) for the comparisons of (b) DSS vs Control and (c) Cur-Ant NPs vs DSS. The analysis categorizes the DEGs into Biological Process (BP), Cellular Component (CC), and Molecular Function (MF). The x-axis represents the gene count.

**Figure S7. Further Analysis of Gut Microbiota Alterations in DSS-Induced Colitis Mice, Related to Figure 9.** (a, b) Additional alpha diversity metrics for the gut microbiota: (a) Faith’s Phylogenetic Diversity (PD) index and (b) Pielou’s evenness index. (c) Principal Coordinates Analysis (PCoA) score plot illustrating the beta diversity of the gut microbiota. The variation explained by PCoA1 and PCoA2 is 22.2% and 14.22%, respectively (PERMANOVA *p* = 0.001). (d–f) Box plots showing the relative abundance of specific bacterial taxa significantly altered by DSS and modulated by Cur-Ant NPs: (d) family S24-7 (Log10 scale), (e) genus Akkermansia (%), and (f) genus Lactobacillus (Log10 scale). Data in (a, b) are presented as mean ± SD. Data in (d–f) are presented as box plots overlaid with individual data points (n = 5–6 mice per group). Statistical significance was determined by one-way ANOVA. **p* < 0.05, ***p* < 0.01, ****p* < 0.001. ns indicates no significant difference.


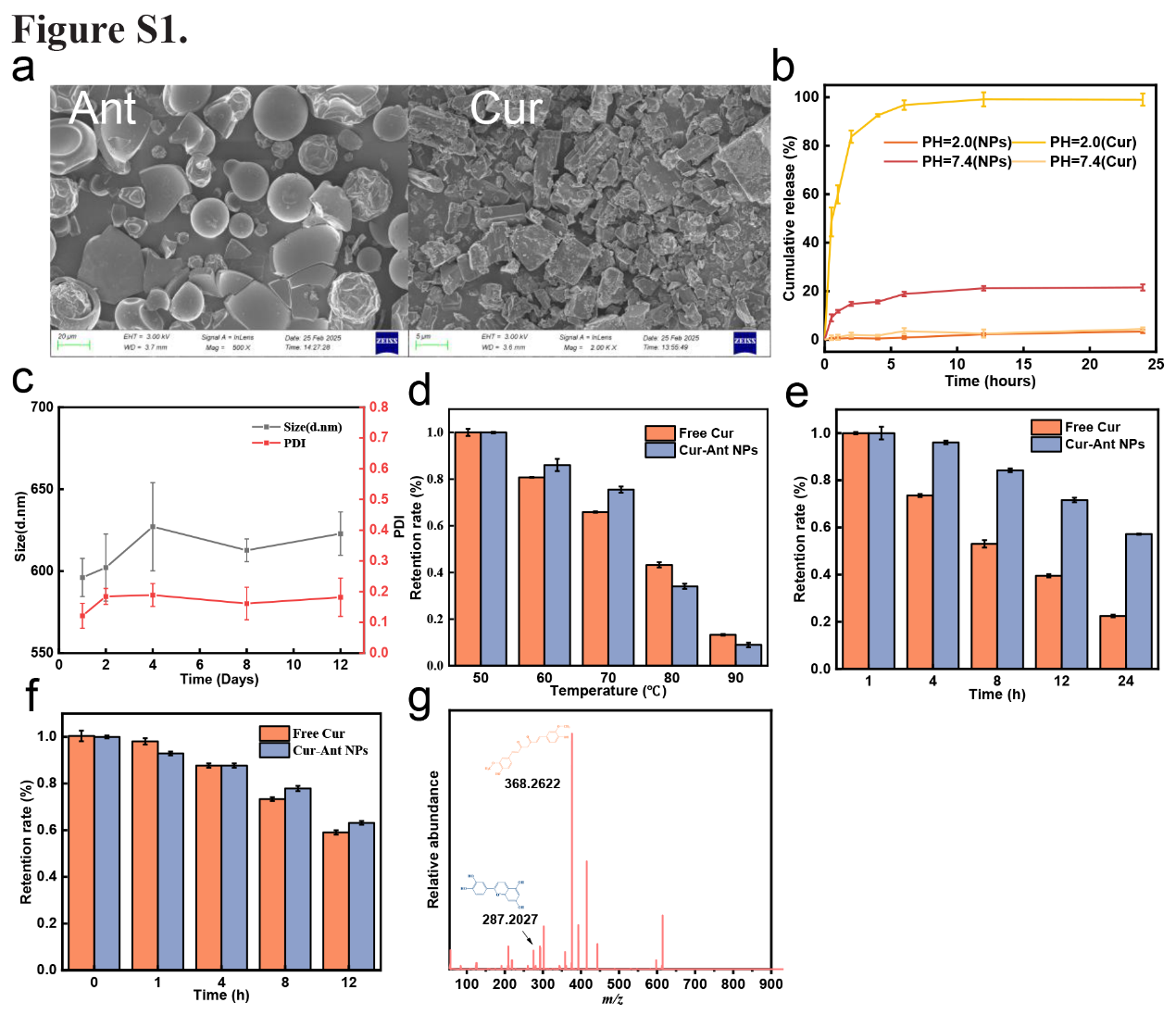


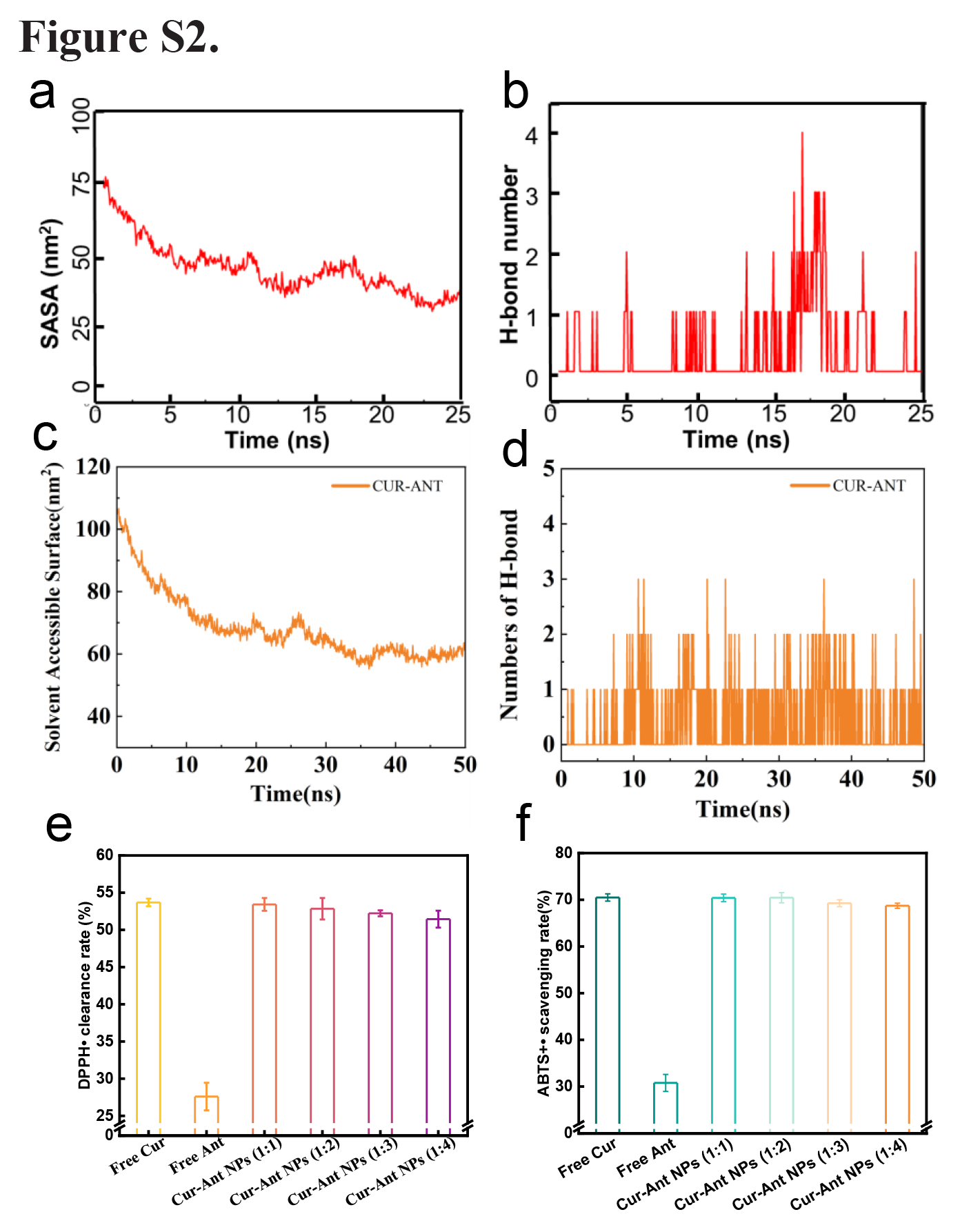


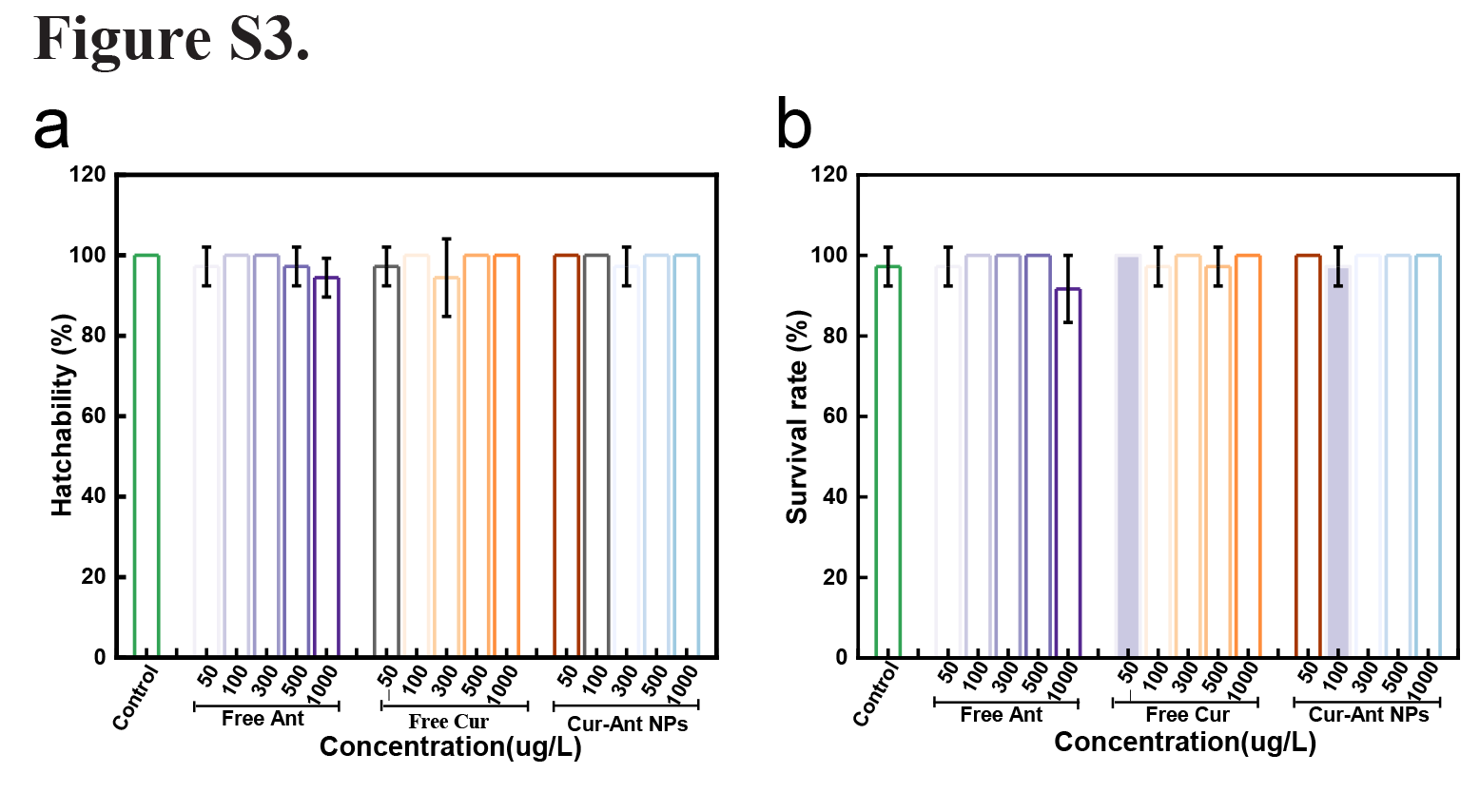


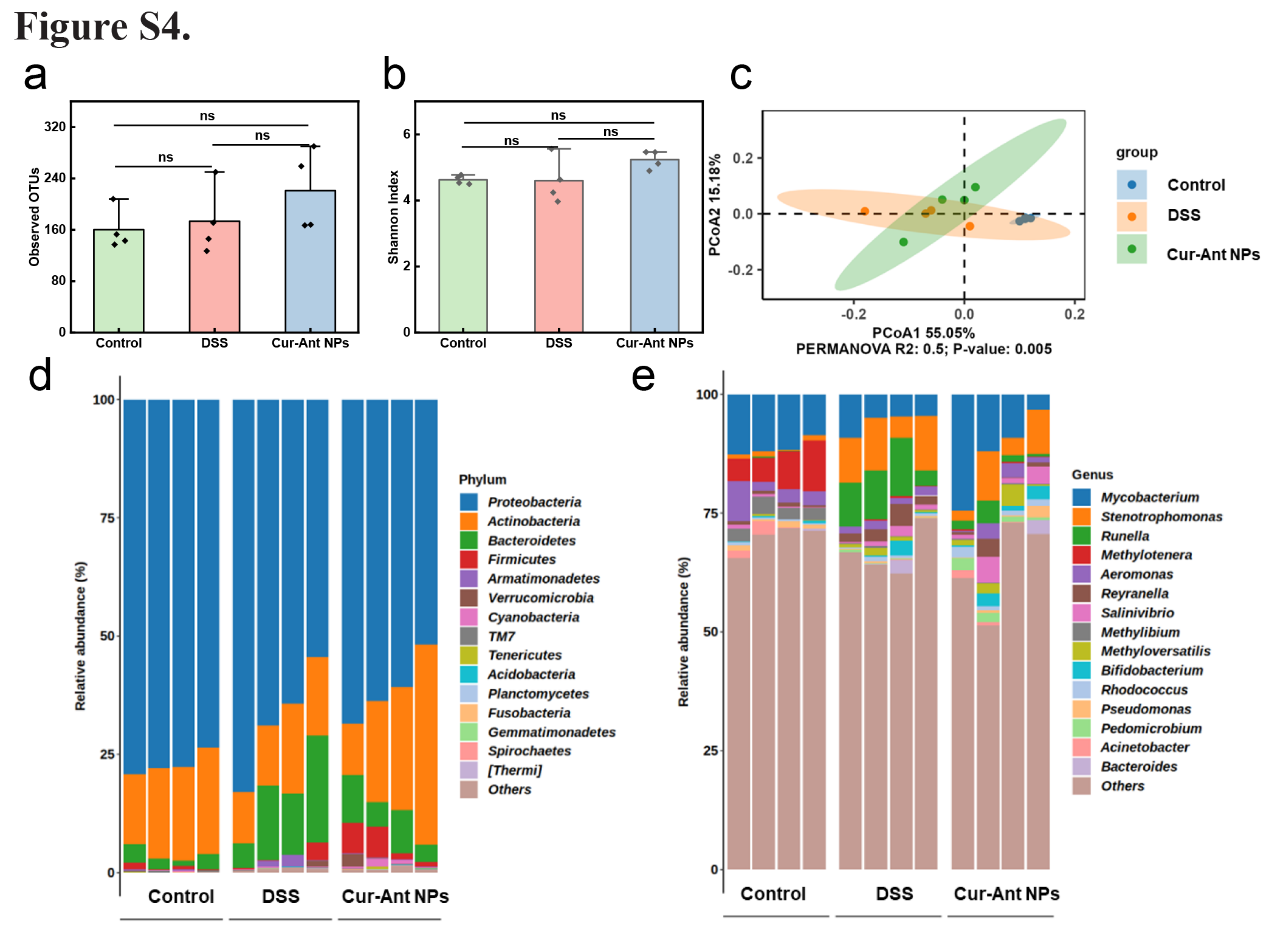


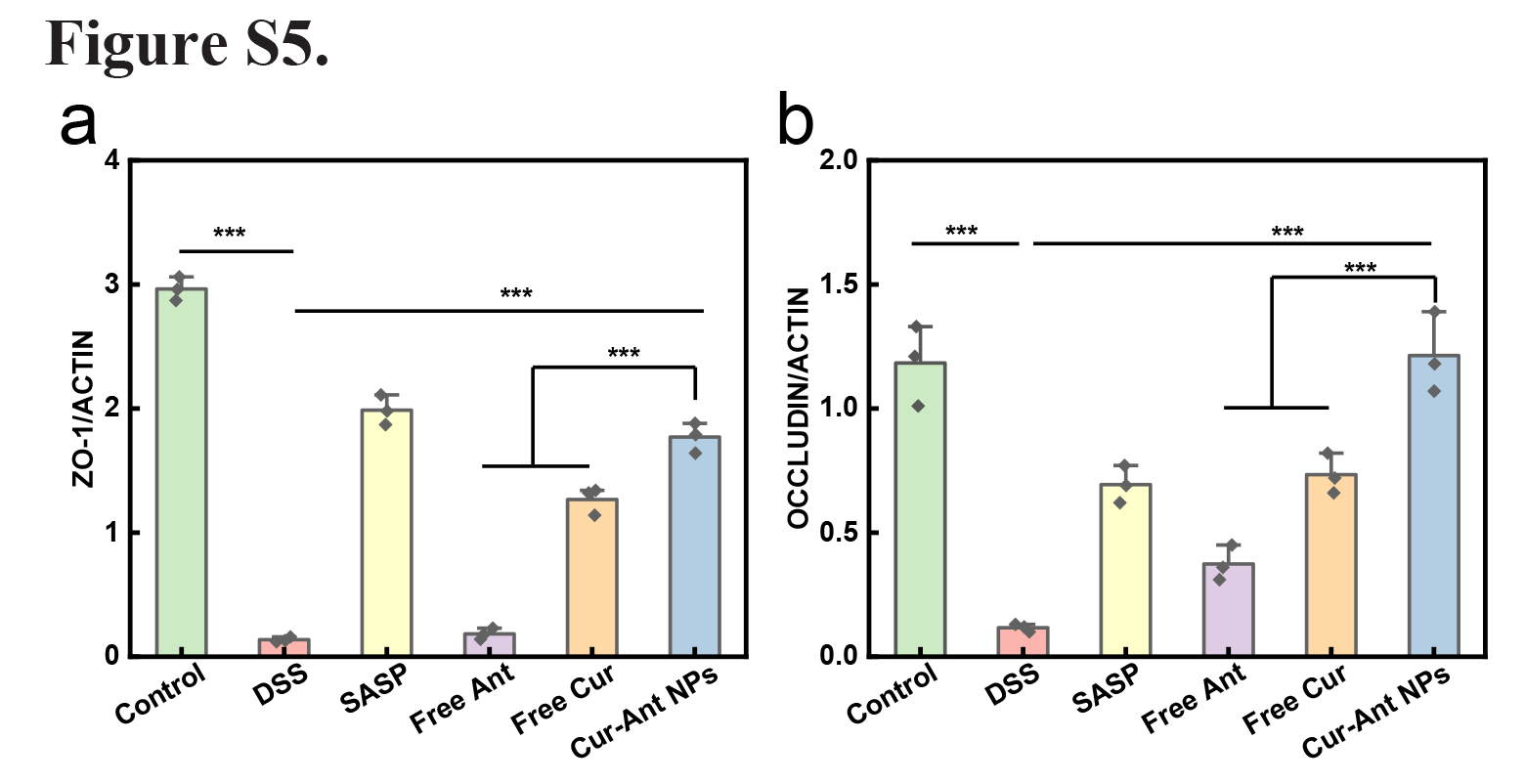


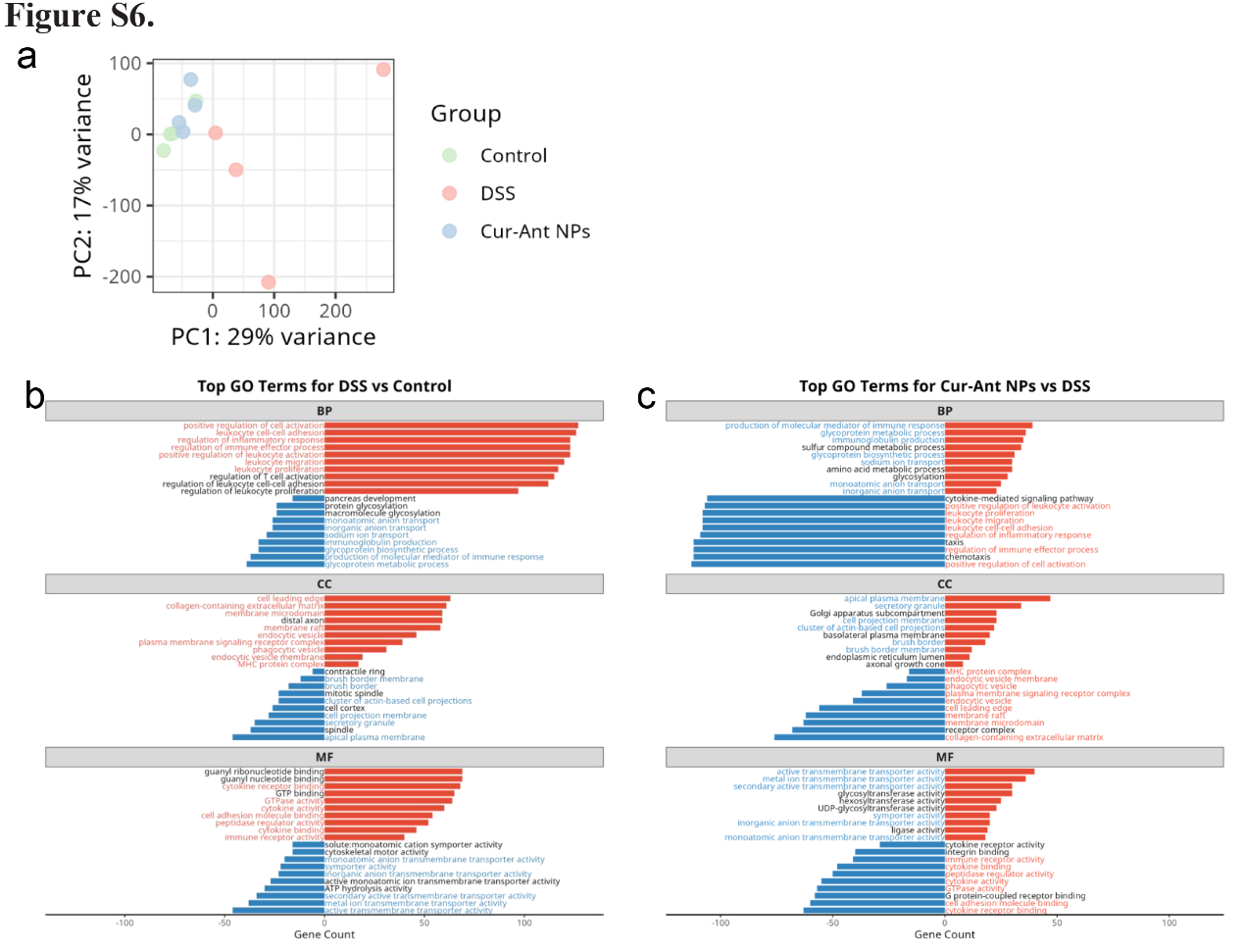


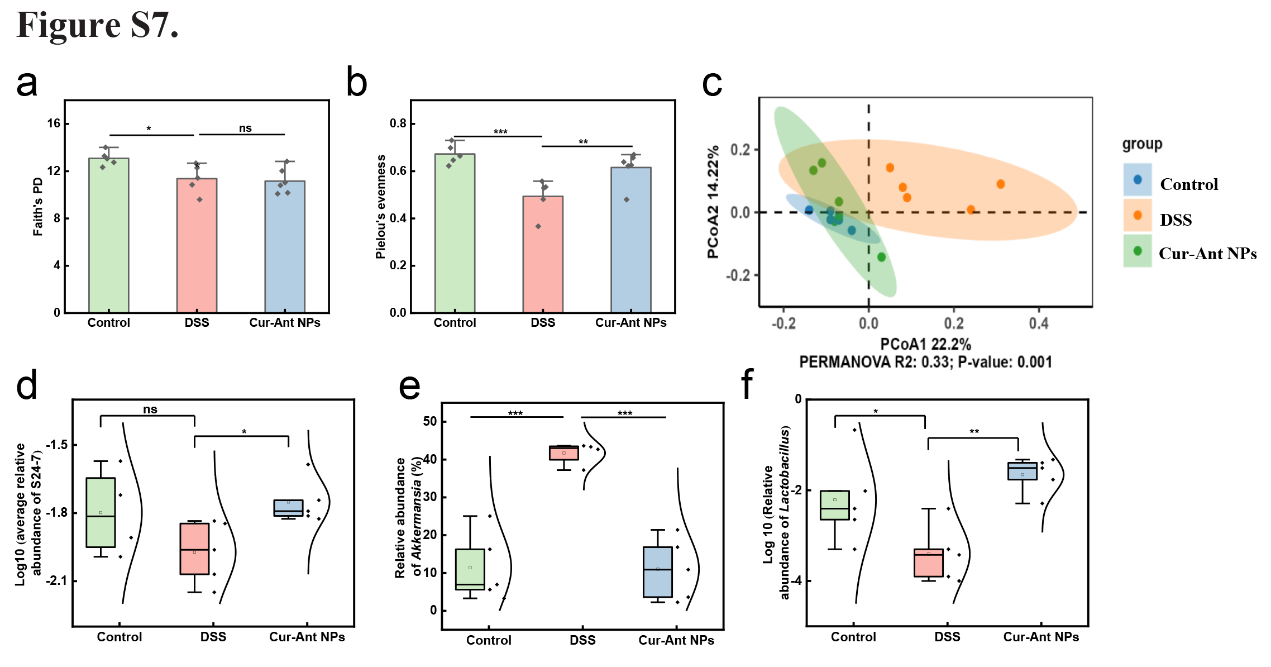

Supplement: Multimedia component 1 [file mmc1.docx]
